# Joint representation of color and form in convolutional neural networks: A stimulus-rich network perspective

**DOI:** 10.1371/journal.pone.0253442

**Published:** 2021-06-30

**Authors:** JohnMark Taylor, Yaoda Xu

**Affiliations:** 1 Department of Psychology, Vision Sciences Laboratory, Harvard University, Cambridge, MA, United States of America; 2 Department of Psychology, Yale University, New Haven, CT, United States of America; University of Engineering & Technology, Taxila, PAKISTAN

## Abstract

To interact with real-world objects, any effective visual system must jointly code the unique features defining each object. Despite decades of neuroscience research, we still lack a firm grasp on how the primate brain binds visual features. Here we apply a novel network-based stimulus-rich representational similarity approach to study color and form binding in five convolutional neural networks (CNNs) with varying architecture, depth, and presence/absence of recurrent processing. All CNNs showed near-orthogonal color and form processing in early layers, but increasingly interactive feature coding in higher layers, with this effect being much stronger for networks trained for object classification than untrained networks. These results characterize for the first time how multiple basic visual features are coded together in CNNs. The approach developed here can be easily implemented to characterize whether a similar coding scheme may serve as a viable solution to the binding problem in the primate brain.

## Introduction

Natural visual experience comprises a juxtaposition of different visual features, such as an object’s color, position, size, and form, with the form features including both simple form features such as local orientations and contours, and the complex form features including global shape and texture, which often define an object’s identity. To recognize an object under different viewing conditions, our visual system must successively reformat and “untangle” the different features to make object identity information explicitly available to a linear readout process in a manner that is tolerant to variations in other features, an ability that has been hailed as the hallmark of primate high-level vision [1, 2].

Meanwhile, our interaction with the world often involves objects with uniquely defined features, such as grabbing the blue pen on the desk. How would an object representation that sheds all its identity-irrelevant features support our ability to interact with specific objects? One possibility is that different visual features are initially processed separately and are bound together via attention (i.e., Feature Integration Theory [3]. Despite decades of neuroscience research, the coding mechanism for such a binding process remains unknown, with existing proposals facing various challenges. For example [4], proposed that neurons coding for different features of the same object could engage in synchronous oscillations, serving as a binding signal, but it is unclear how such a signal would be generated and read out [5]. Alternatively, there might exist neurons that encode particular feature conjunctions; however, this view collides with the problem of “combinatorial explosion”: there are more possible feature conjunctions than neurons in the brain. Given that the tuning of neurons is affected by a diverse set of mechanisms, including feedforward activation, lateral inhibition, and feedback connections, a step towards understanding how neurons encode feature combinations would be to understand how each of these factors independently contribute to the conjunctive coding of visual features, but this is challenging due to the complexity of the primate visual system.

Recently, convolutional neural networks (CNNs) have achieved human-level object recognition performance [6–9]. Specifically, these CNNs have been trained to disregard identity-irrelevant object features to correctly identify objects across different viewing conditions, thereby forming transformation-tolerant visual object representations much like those in high-level primate vision. In both human fMRI and monkey neurophysiological studies, representations formed in lower and higher layers of the CNNs have been shown to track those of the primate lower and higher visual processing regions, respectively [10–16].

Although CNNs are fully computable and accessible, they are extremely complex models with thousands or even millions of free parameters. Consequently, despite their success in object recognition, the general operating principles at the algorithmic level [17] that enable CNNs’ success remain poorly understood (e.g., [18]). This includes understanding how different types of visual features are represented together in CNNs during the course of visual processing. Several studies have examined how individual features are encoded in CNNs, with some finding that coding for object identity-irrelevant features increases in higher CNN layers [2, 19]. Additional approaches to understanding internal CNN representations (summarized in [9, 20]) include synthesizing images that maximally drive individual CNN units (e.g., [21]), ablating sets of units and examining how this impairs network performance (e.g., [22]), and using principal components analysis to visualize how different features are encoded in a given layer (e.g., [23]). Of relevance to the present work, several studies have reported the color encoding characteristics of CNN units [20, 23–25]. Of these four studies, [23, 24], and [20] examine color coding, but do not examine how color is jointly coded with form. [25] examines this issue, but only do so for a small number of units in a single CNN. No study to date to our knowledge has examined how combinations of color and form features are jointly encoded across entire populations of CNN units.

Because CNNs are not trained to interact with specific objects but simply to produce the correct object labels at the end of its processing, it is possible that different features are initially encoded in an entangled, intermingled fashion, and are gradually separated, with object identity information gradually made more explicit and independent of other visual features over the course of processing [1]. Alternatively, CNN architecture and training for object recognition may automatically give rise to *interactive*, rather than independent, coding of object features in later stages of processing, rendering unnecessary a separate binding operation to encode the relationship between independent features. This could constitute a novel binding mechanism that has not been considered before in neuroscience research. Thus, studying how CNNs jointly encode different object features during the course of visual information processing is not only timely in its own right, but also provides us with a unique opportunity to gain insight into the potential computational algorithm that a successful object recognition system may use to code different object features together. Moreover, the wiring of most CNNs is restricted to feedforward connections, making it tractable to isolate how various aspects of conjunctive tuning may arise from feed-forward processing alone. Equally importantly, given that the internal representations of CNNs are fully image computable and freely inspectable, CNNs provide ideal testing grounds for developing analysis methods to study feature coding across an entire processing hierarchy and with a large number of objects, and generating hypotheses that can be tested in biological visual systems. Finally, CNNs are trained for a well-defined task (typically object recognition), making it possible to examine how task demands shape the joint representation of multiple features.

In this study, we examined how an object’s color and form may be coded together during visual processing in CNNs. Specifically, we devise a new metric that captures the extent to which color and form are encoded in an interactive manner—that is, the extent to which the coding for one feature varies across values of the other. Broadly, our method involves examining whether the similarity structure of different colors varies across different object form features. Our method is entirely inspired by neuroscientific studies of primate vision. For example [26], examine whether shape tuning varies across colors (a direct analogy of our analysis) [27], examine whether color and form tuning is additive in IT neurons (which corresponds to an orthogonal representation in our scheme) [28], examine how color tuning varies across different shapes in the macaque color-sensitive patches (once more, a direct analogy of our analysis), and [29] studies whether color and form are encoded independently versus interactively in human retinotopic cortex. These studies have varied in their conclusions, with some finding evidence for independent coding and others interactive coding, depending on the brain region studied and the stimuli used. Our study thus extends and bridges the investigation of joint color/form representation from the primate brain to CNNs, and doing so in a comprehensive, stimulus-rich way that would be challenging in neural experiments: whereas it would be challenging to comprehensively study every stage of the hierarchy in the primate brain, using both naturalistic and artificial stimuli, the inspectability of CNNs renders this much more feasible. With this approach, we found that coding for color and form becomes increasingly interactive throughout CNN processing. These results thus characterize, within a novel population-coding framework, how multiple visual features are encoded together in CNNs. The approach developed here can be easily implemented to characterize whether the primate brain may use a similar coding scheme to solve the binding problem.

## Results

In this study, we examined in detail how color and naturalistic object form features may be represented together in five CNNs trained for object recognition using ImageNet [30] images. These CNNs, chosen for their high object recognition performance, architectural diversity, and prevalence in the literature, included AlexNet [31], VGG19 [32], GoogLeNet [33], ResNet-50 [34], and CORNet-S [35]. Specifically, AlexNet was included for its relative simplicity and prevalence in the literature. VGG19, GoogLeNet and ResNet-50 were chosen based on their high object recognition performance and architectural diversity. Both AlexNet and VGG19 have a shallower network structure, whereas GoogLeNet and ResNet-50 have a deeper network structure. CORNet-S is a shallow recurrent CNN designed to approximate the structure of the primate ventral visual pathway, and exhibits high correlation with neural and behavioral metrics. This CNN has recently been argued to be the current best model of the primate ventral visual regions [35]. These networks differ in both their architecture, and in some cases, their training regimes; specifically, for training data augmentation AlexNet, VGG19 and ResNet-50 use cropping, horizontal flips, and RGB adjustments (simulating changes in lighting), whereas CORNet-S only uses cropping and horizontal flips, and GoogLeNet only employs cropping. We sampled between 6 to 9 layers in each of these CNNs (Table 1). These layers included the first layer, layers demarcating the boundary between meaningful “segments” of the network, the penultimate layer and the last layer (which is the classification layer), and any other fully-connected layers. The layers were also chosen in such a manner as to sample the network as evenly as possible, and to at least roughly equate the number of layers extracted from each network. In a control analysis, we found that our sampled layers capture the overall processing trajectory of the network and that the trajectory does not change with the types of layers sampled, as long as they are adjacent to each other in the processing pipeline (S1 Fig). Although fully-connected layers (including the classification layer) differ from early layers in the network in that they do not follow a weight-sharing constraint over space, past work has found that they encode not just information about object category membership, but also information about features such as shape, position, spatial frequency, and size [19, 36], making it appropriate to examine how they jointly encode the features of shape and color at the end of CNN visual processing.

**Table 1:** The five CNNs included in the present study and the layers sampled in each CNN (PyTorch labels given).

Network	Layers Used
(Layers Used/Total Layers)	
AlexNet (8/25)	*Features*: (0) Conv2d, (3) Conv2d, (6) Conv2d, (8) Conv2d, (10) Conv2d
	*Classifier*: (1) Linear, (4) Linear, (6) Linear
CORnet-S (7/42)	*V1*: (conv1) Conv2d, (nonlin2) ReLU,
	*V2*: (nonlin3) ReLU
	*V4*: (nonlin3) ReLU
	*IT*: (nonlin3) ReLU
	*Decoder*: (avgpool) AdaptiveAvgPool2d, (linear) Linear
GoogLeNet (6/144)	(conv1) BasicConv2d, (maxpool2) MaxPool2d,
	(maxpool3) MaxPool2d, (maxpool4) MaxPool2d,
	(avgpool) AdaptiveAvgPool2d, (fc) Linear
ResNet-50 (6/177)	(conv1) Conv2d, (layer1) (2) (relu) ReLU,
	(layer2) (3) (relu) ReLU, (layer3) (6) (relu) ReLU,
	(avgpool) AdaptiveAvgPool2d, (fc) Linear
VGG19 (9/47)	*Features*: (0) Conv2d, (4) MaxPool2d, (9) MaxPool2d,
	(18) MaxPool2d, (27) MaxPool2d, (36) MaxPool2d,
	*Classifier*: (0) Linear, (3) Linear, (6) Linear

**Fig 1:**
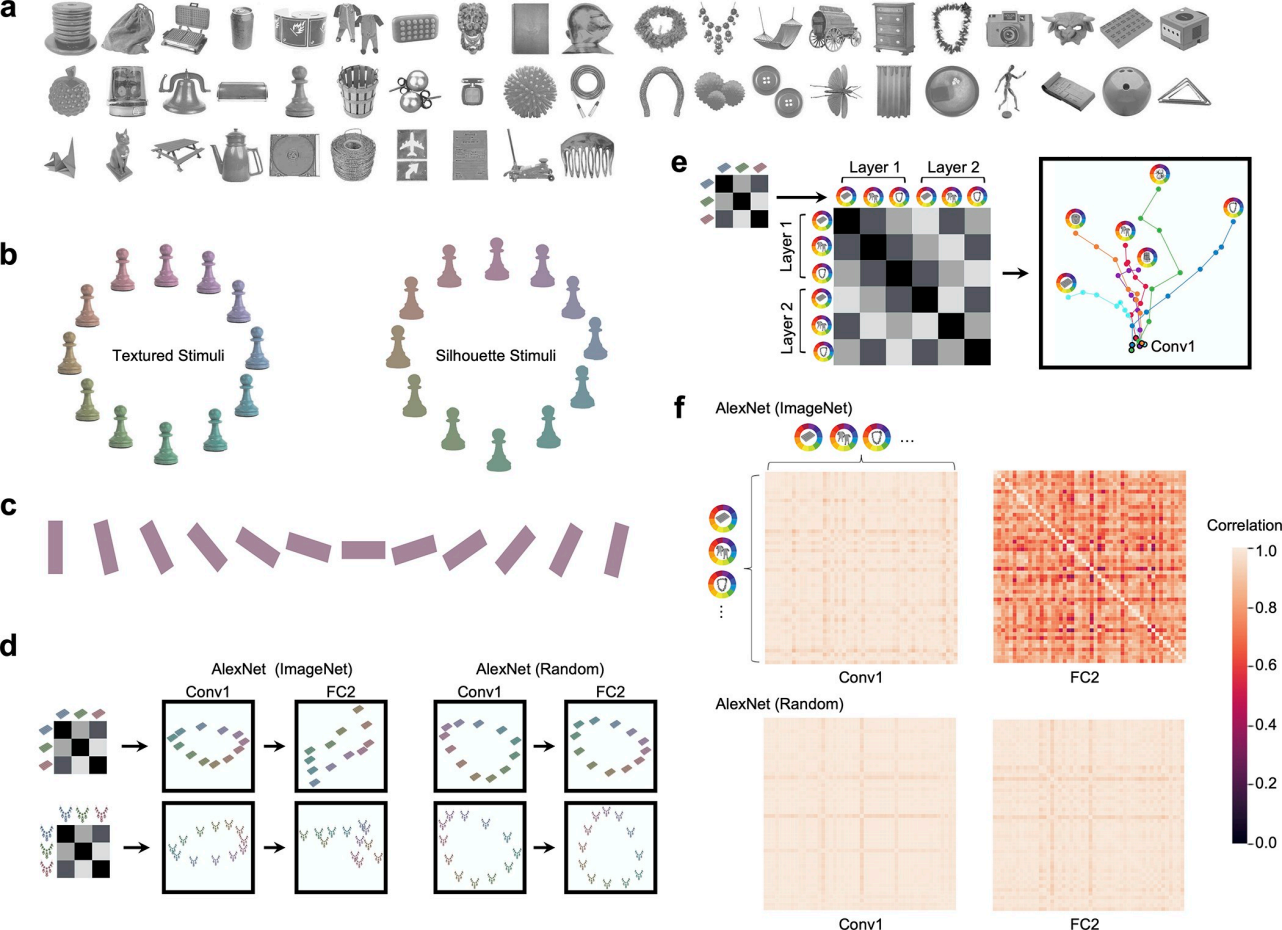
Stimuli used and example color space characterization using RSA and MDS. **a.** The 50 objects included in the main stimulus set, chosen from an initial set of 500 objects to maximize their mean pairwise pattern dissimilarity in AlexNet FC2. **b.** The 12 isoluminant and iso-saturated colors (based on the CIELUV color space) and the two versions of the object shapes used in the main analysis. Objects appeared either with their original textures preserved (“Textured Stimuli”), or as uniformly shaded silhouette stimuli (“Silhouette Stimuli”). **c.** The 12 oriented bar stimuli used in a control analysis. **d.** An illustrative color similarity matrix for a given object (left-most column) and actual MDS plots showing the representational structure of two example objects each in the 12 colors calibrated in CIELUV color space from Conv1 and FC2 of AlexNet trained with ImageNet (2nd and 3rd columns from left). Color space correlations were first obtained from each object in the 12 colors (3 colors were illustrated here) in a given layer to construct a color similarity matrix for that layer. This similarity matrix was then placed on a 2D space using MDS. While the similarity spaces of these objects have a similar elliptical pattern at the beginning of the trained AlexNet, by the end of processing the color spaces of these objects are substantially different both from each other and from those at the beginning of the processing. By contrast, in a version of AlexNet with randomized weights (two right columns), the color spaces of both objects remain roughly similar at both the beginning and end of processing, as shown by the similar arrangement of the colors of each object. **e.** An illustrative color space similarity matrix (left) and an actual MDS plot showing the color spaces of six example objects over the course of processing in AlexNet (right). Color spaces were computed separately for each of these objects in each sampled layer of AlexNet (illustrative color space depicted by the small matrix on the left), and the resulting color spaces (only three objects and two layers illustrated here) were correlated with one another to construct a color space similarity matrix (note this is a second order correlation matrix, different from the color similarity matrix illustrated in d). This similarity matrix was then placed on a 2D space using MDS. Each dot in the MDS plot represents the color space of a given object at a given layer, where the distance between two dots reflects the similarity between two color spaces. Each trajectory traces the color space of a given object. The dot corresponding to the initial layer has a black outline, and the dot corresponding to the final layer is marked by a picture of the object for that trajectory. While the color spaces of different objects are initially very similar, by the end of processing they have substantially diverged. **f.** Actual representational similarity matrices showing the pairwise color space similarity for each pair of objects in both the first and penultimate layers of AlexNet, in both its trained and untrained variants.

We used representational similarity analysis (RSA, [37]) to characterize how color and form information is represented together in these networks. For most analyses, we studied a set of 50 objects (chosen from a set created by [38]), each colored in 12 colors that were calibrated to equate their mean luminance and saturation in the CIELUV color space before being converted back to RGB as inputs to each network (Fig 1A and 1B). Equating luminance was necessary to ensure that any results were truly driven by color-related processing rather than luminance contrast mechanisms, and equating saturation was important for equating the hue variability within each object. Two versions of each object were shown: a textured version, with internal object detail preserved, and a silhouette version with all non-white pixels set to a uniform color, thus comprising a global form contour without internal details (Fig 1B). Examining both the normally-textured objects and the silhouettes allowed us to make more precise inferences about how color and form information interact: while the textured objects differ both by their outline and texture, the silhouettes vary with respect to their outline alone, and so to the extent that any results also hold for the silhouettes, it would demonstrate that these results do not depend on texture differences. Additionally, removing texture details removes much category-diagnostic information from the stimuli, clarifying whether category-specific effects may be driving any results.

Broadly, our analyses examined how color and form information are jointly encoded over the course of processing in CNNs. To examine this question within a population coding framework, we define a color space as the similarity profile of a set of colors for a given stimulus in a given representational context (e.g., for a given object and CNN layer). To the extent that color spaces are invariant across objects, color and form information can be said to be independent or orthogonal; to the extent that they differ, they can be said to be interactive or entangled. Several analyses were performed on these color spaces. The specific analyses involved (1) comparing the color spaces across objects within each layer, to determine whether color and form are encoded independently versus interactively in that layer, and examining how this is affected by variations in stimuli, analysis parameters, and network training regime; (2) quantifying the magnitude of the representational distances among the different colors of an object, to assess the overall strength of color representation throughout processing (3) comparing the color space for each object across layers, to determine how color information for each object is transformed over the course of processing; (4) examining whether color space differences across objects are preserved across layers, and (5) whether the form similarity of two objects predicts their color space similarity. While we use MDS plots for visualization purposes and to build intuition, all conclusions are grounded in subsequent statistical analyses. In cases where we compared coding over the course of CNN processing or compared coding at the beginning and end of CNN processing, the first through *penultimate* layers, or the first and *penultimate* layers, were used, respectively. Although the final sample layer (i.e., the classification layer) contains feature representation as stated earlier, given that this layer is constructed to pool the processed object information together to assign category labels rather than to further process object information, choosing the penultimate layer here would better reflect the state of visual information representation at the end of CNN processing. To our knowledge, these analyses provide the first in-depth and comprehensive network-based description of how colors and form are coded together in CNNs.

### Visualizing color space representation across objects and CNN layers

As our primary analysis, we applied RSA to examine the extent to which coding for color varies across objects, and the extent to which the magnitude of this variability changes across CNN layers. As an initial exploratory analysis, we visualized how the color spaces of two example objects may differ at the beginning and end of processing in AlexNet, examining both a trained and untrained version of the network (Fig 1D). Specifically, we extracted the activation patterns for the 12 colors of these two objects (textured versions) from the first and the penultimate layers of AlexNet (Conv1 and FC2). Within each layer, we performed all pairwise Pearson correlation among the 12 patterns to create a representational similarity matrix (RSM). After subtracting each correlation from 1 to convert the similarities to dissimilarities, we used multidimensional scaling (MDS) [39] to visualize the resulting representational dissimilarity space placed on 2D space, with a closer distance between a pair of colored objects indicating more similar representations (Fig 1D). In the version of AlexNet trained on ImageNet, color appeared to be coded similarly at the beginning of the network for these two objects, as reflected by the similar elliptical shape of the color spaces; by the end of processing, however, the color spaces of these two objects seem to differ substantially, both from each other and from their color spaces at the beginning of processing. By contrast, in an untrained version of AlexNet the two objects had similar, ellipse-shaped color spaces at both the beginning and end of processing.

To generalize from these two objects and examine how the color space for different objects might diverge over layers, we visualized the evolution of the color spaces of six example objects over the course of processing in the trained version of AlexNet (Fig 1E). To do this, for each object (textured versions) and for each sampled layer of AlexNet, we first constructed a “color space” RSM by performing all pairwise Pearson correlations of the patterns associated with the 12 different colors of that object. We vectorized the off-diagonal values of this RSM to create a “color space” vector. Next, we performed all pairwise correlations of these “color space” vectors across objects and layers to form a “color space similarity” RSM that quantifies how similarly color is coded in different objects and layers; finally, each value was subtracted from 1 to convert the matrix to a dissimilarity matrix. We then used 2D MDS to visualize the resulting representational similarity space, where each dot represents the color space of a given object at a given layer (i.e., the similarity profile among the different colors of the object at that layer), and the distance between two dots reflects how similarly color is coded in those two spaces (Fig 1E). In these objects, color appeared to be initially coded in a very similar manner (as reflected by the dense clustering of the bold-outlined dots representing the different color spaces in the initial layers of processing), but color coding increasingly diverged as processing proceeded in the network (as reflected by the separation of the dots at the end of processing, indicated by the object icons next to the dots). In other words, over the course of processing, color coding for each object both increasingly differed from the color coding for other objects, and from the color coding of that object at the beginning of processing. Fig 1F shows the full RSM of the color space similarities among every pair of objects for both a trained and an untrained version of Alexnet. As an initial observation, the color spaces of all objects appear to be very similar to each other early in processing for both trained and untrained AlexNet, but by the end of processing the color spaces associated with the different objects have diverged for the trained, but not the untrained, version of AlexNet.

As a control analysis, we also performed a similar visualization for three objects, but sampling from *all* layers of AlexNet to confirm that our sampled layers indeed capture the overall processing trajectory of the network (S1 Fig) and that the trajectory does not change with the types of layers sampled, as long as they are adjacent to each other in the processing pipeline.

### Quantifying color space differences across objects within a CNN layer

**Fig 2:**
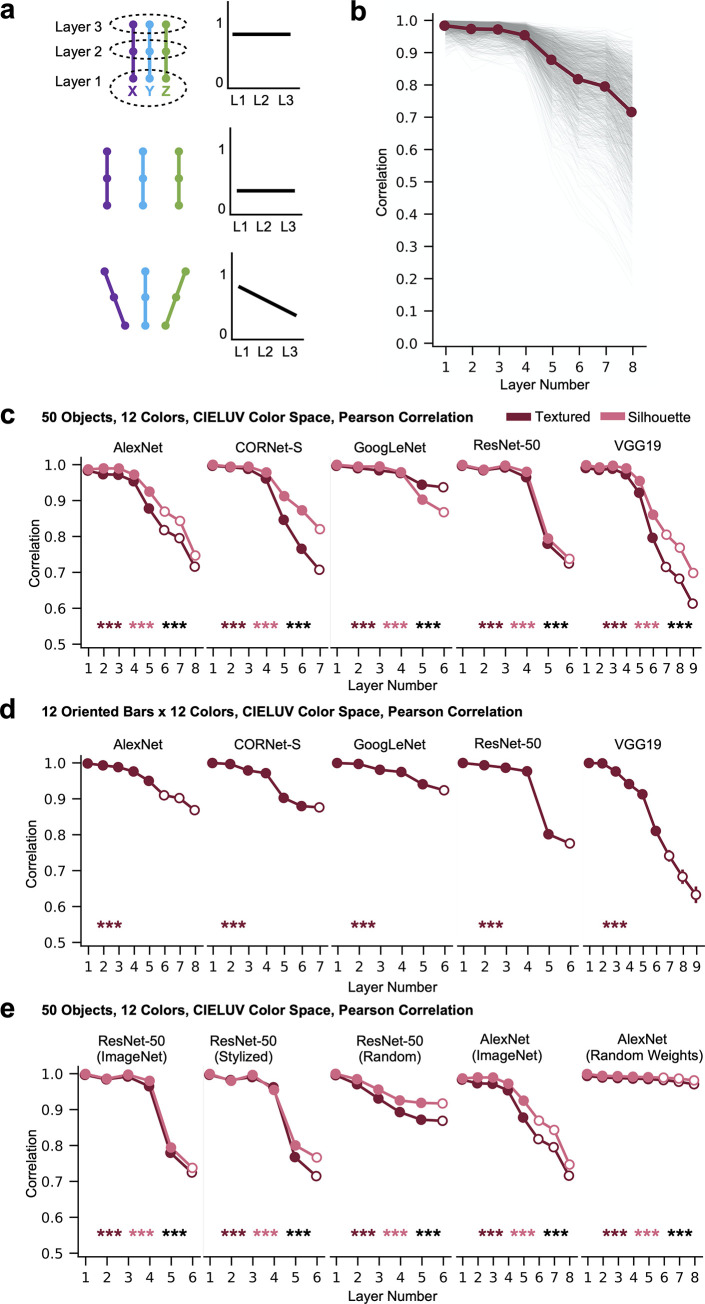
Color space representation across objects within a CNN layer. a. A schematic illustration of three possible scenarios. In each scenario, the left figure illustrates the color space transformation of three objects in three hypothetical CNN layers, with each colored dot depicting a color space structure of an object at a CNN layer and each trajectory depicting an object. The right figure in each scenario illustrates how the mean pairwise correlation of all object color spaces for a given layer changes across layers. In the first scenario, the color spaces of the three objects remain relatively similar within each layer throughout processing. In the second scenario, they are dissimilar within each layer throughout processing. In the third scenario, they are similar in the first layer, but become dissimilar in later layers. b. The pairwise color space similarity for every pair of textured objects in each layer of Alexnet for the full set of 50 objects in the 12 colors calibrated in CIELUV color space. The color space structure of each object is measured with Pearson correlation (this also applies to c-e below). Each thin grey line is the color space similarity for a single pair of objects, with the bold line showing the mean across all pairs. c. The mean pairwise color space similarity for each sampled layer of each of the five CNNs, for the full set of 50 objects in the 12 colors calibrated in CIELUV color space. Results are shown for both the textured objects (in maroon) and the silhouette objects (in pink). Fully-connected layers are marked by hollow circles and other types of layers sampled are marked by solid circles. Linear regression was used to measure the downward trend of the correlation values across layers for each object pair. The mean of the resulting slopes (one slope per object pair) were tested against zero for each of the two versions of the objects, and the difference between the two sets of slopes was also tested (with significance levels marked by maroon and pink asterisks, respectively, for each of the two versions against zero, and by black asterisks for the differences between the two versions). In all cases, mean pairwise color space similarity decreases over the course of processing, with this decline being greater for the textured than for the silhouette objects, with the exception of GoogLeNet. d. Mean color space similarity across the 12 oriented bar stimuli in the 12 colors calibrated in CIELUV color space. Even in these minimally simple form stimuli, mean pairwise color space similarity decreases over the course of processing. e. Mean color space similarity across the 50 objects in the 12 colors calibrated in CIELUV color space in CNNs with different training regimes. Comparisons are made among ResNet-50 trained with the original ImageNet images, trained with stylized ImageNet images, and with 100 untrained random-weight initializations of the network. Comparisons are also made between AlexNet trained with the original ImageNet images, and with 100 untrained random-weight initializations of the network. Averaged results are shown from the 100 untrained versions of each network. The untrained networks exhibit a much smaller decline in their mean pairwise colorspace correlation across objects than the trained networks. *** *p* < .001.

Next, we quantified and further explored the divergence in object color spaces over the course of processing that we qualitatively observed in the previous section. To quantify the color space divergence among different objects within a layer and over the course of processing, we computed the averaged pairwise color space vector correlations for the 50 objects in each layer of each CNN, and for both the textured and silhouette stimuli. This between-object color space similarity measure is a correlation-of-correlations metric: it captures the extent to which color coding varies across form features, with the first order correlation capturing the structure of the color space for each individual object and the second-order correlation capturing how the color space structure varies across the different objects, with lower correlation indicating more interactive color and form coding. For the entire set of 50 objects, several patterns of results, shown in Fig 2A, could be possible: the color spaces of different objects might be highly similar in every layer, they might be highly dissimilar in every layer, or they might begin similar to each other, but diverge over the course of processing.

Fig 2B shows the color space correlation between every pair of objects, as well as the mean of these correlations, for the textured version of the objects run through AlexNet. We observe a reliable decrease in the color space correlation of different objects as processing proceeds. To generalize these findings across networks and quantify statistical significance, Fig 2C depicts the mean between-object color space correlation within every layer of every network as well as how the mean changes across layers, for both the textured and silhouette versions of the objects. In all CNNs, and in both stimulus conditions (textured and silhouette), the mean color space correlations were high in lower layers but then significantly decreased from mid to high CNN layers.

As an additional measure to ensure that there were no lurking differences in the “baseline” between-object color space correlations for different models and training regimes, for the trained version of AlexNet, the untrained version of AlexNet, and the trained version of GoogLeNet, we performed a resampling procedure in which we randomly shuffled the labels of the colors within each object (independently for each object) and computed the mean pairwise color space correlations among all pairs of objects; this was done one hundred times, and the resulting color space correlations were averaged. The resulting mean correlations were near zero in every single layer of all three tested models (highest r = .0005; see S1 Table for the full results). We thus found no evidence that spurious differences among the different model architectures, layer types, or training regimes could have driven our main results in the absence of genuine color space correlations among objects.

We further quantified, using regression analysis, whether this mean between-object color space similarity significantly declines over the course of processing, and whether this decline varies significantly between the textured and silhouette versions of the stimuli (see Methods for analysis details).

We measured the decrease in the correlation values over layers by testing the presence of a negative slope. We note that our conclusions do not require a purely monotonic progression of the correlation values. Nevertheless, the presence of a negative slope would still signal any presence of a negative linear trend. We found an overall significantly negative slope across all the layers for each of the networks tested (see the asterisks marking the significance level of the slope at the lower part of each plot). Coding of color thus remained relatively similar across objects in lower layers but then became increasingly different from mid to high CNN layers, reflecting what is depicted in Figs 1C and 2A bottom panel. Since this increase in interactive tuning occurred even for the silhouette stimuli, it did not depend on the internal texture features of the stimuli, and can occur with respect to global form features alone. That being said, for most of the networks, the textured stimuli did exhibit a greater drop in their color space similarity over the course of processing than the silhouette stimuli, with the exception of GoogLeNet, suggesting the existence of greater interactive coding for texture features above and beyond global form features alone.

To ensure that these results did not arise due to the particular similarity metric used in constructing the RSMs (i.e., Pearson correlation), we repeated the same analysis using Euclidean Distance to measure the similarity between the different colors of each object; Pearson correlation was still used to measure the second-order similarity *between* the color spaces (S2A Fig). Additionally, to ensure that our results did not depend on the human-based color space we used (i.e., CIELUV color space), we repeated the same analysis, but using stimuli where the saturation and luminance were equated according to a new color space we constructed, “synthetic HSV”, which was not based on human psychophysical measurements, but was constructed to parametrize the concepts of luminance and saturation for CNNs (S2B Fig; see also Methods). As with the stimuli tuned in CIELUV color space, images were converted back to RGB space prior to running them through the networks. For both manipulations, the results remained qualitatively similar: color space correlations among different objects began high in early layers, and dropped significantly in later layers in all conditions.

To what extent do these results depend on this specific stimulus set? For example, it is possible these results have arisen due to the objects subtending different areas of space. Some stimuli, like the top hat, covered large areas, while other stimuli, like the necklace, covered small areas (Fig 1A). This could have activated different numbers of units in CNN layers and affected how colors are coded for each object. Additionally, it is theoretically possible that results could have been driven by differences in object category rather than form per se, perhaps due to the fact that all networks were trained to recognize objects. To investigate this possibility and to examine whether our results hold even for minimally simple stimuli, we repeated the same analysis (testing how color coding varies based on form) on 12 oriented bars presented in the same 12 colors equated in CIELUV color space as used earlier (Fig 1C). We found the same overall result: as processing proceeds in each network, color coding increasingly differs across different form features (Fig 2D). Thus our results hold for objects equated in their spatial coverage. Moreover, results obtained from complex natural objects can be generalized to simple form stimuli.

Overall, across all conditions we examined, we found a consistent pattern: all CNNs showed near-orthogonal color and form processing in early layers, but increasingly interactive feature coding in higher layers.

### The effect of training on CNN color space representation

The ImageNet images used to train the CNNs studied so far contain real-world objects with natural color-form covariation (e.g., bananas are yellow). Could the interactive color and form coding observed so far in CNNs be driven by such covariation in the training images? To address this question, we compared results from the version of ResNet-50 trained on the original ImageNet images and the version trained on stylized ImageNet images in which the original texture and color of every single image was replaced by the style of a randomly chosen painting, removing the real-world color-form covariation in the natural objects [40]. Interestingly, the version of ResNet-50 trained on stylized images exhibited a significant, steep decrease in their color space correlation over the course of processing (Fig 2E), and the mean slope was slightly, though significantly, steeper than for the version of ResNet-50 trained on ImageNet (*t(1224)* = 3.5, *p* < .001) in the case of the Textured images, with no difference between the two training regimes for the Silhouette images (*t*(1224) = 1.38, *p* = .17). This suggests that the interactive color and form coding observed in CNNs does not rely on the presence of consistent color and form pairing naturally occurring in the training images.

To understand the extent to which the effects we observe may arise due to the intrinsic architecture of the networks versus being a result of object classification training, we examined 100 random-weight initializations of AlexNet and 100 random-weight initializations of ResNet-50, and compared the results with those from the ImageNet image-trained AlexNet and ResNet-50 and the stylized ImageNet image-trained ResNet-50. Results for the 100 random initializations of each network were computed independently, and then averaged together at the final stage (i.e., the between-object color space correlations were averaged). As shown in Fig 2E, while the random networks still exhibited a significant decline in their mean pairwise colorspace correlation across objects, this decline was small, and much smaller than in the corresponding trained version of each network (matched-pairs t-tests; *ps* < .001).

Overall, these results show that the intrinsic CNN architecture is not sufficient to give rise to the large interactive color and form coding observed so far. Training on the object classification task, even with inconsistent pairings of color and form in the object stimuli, appear to play a more significant role in creating such coding.

### Quantifying the strength of color representation

**Fig 3:**
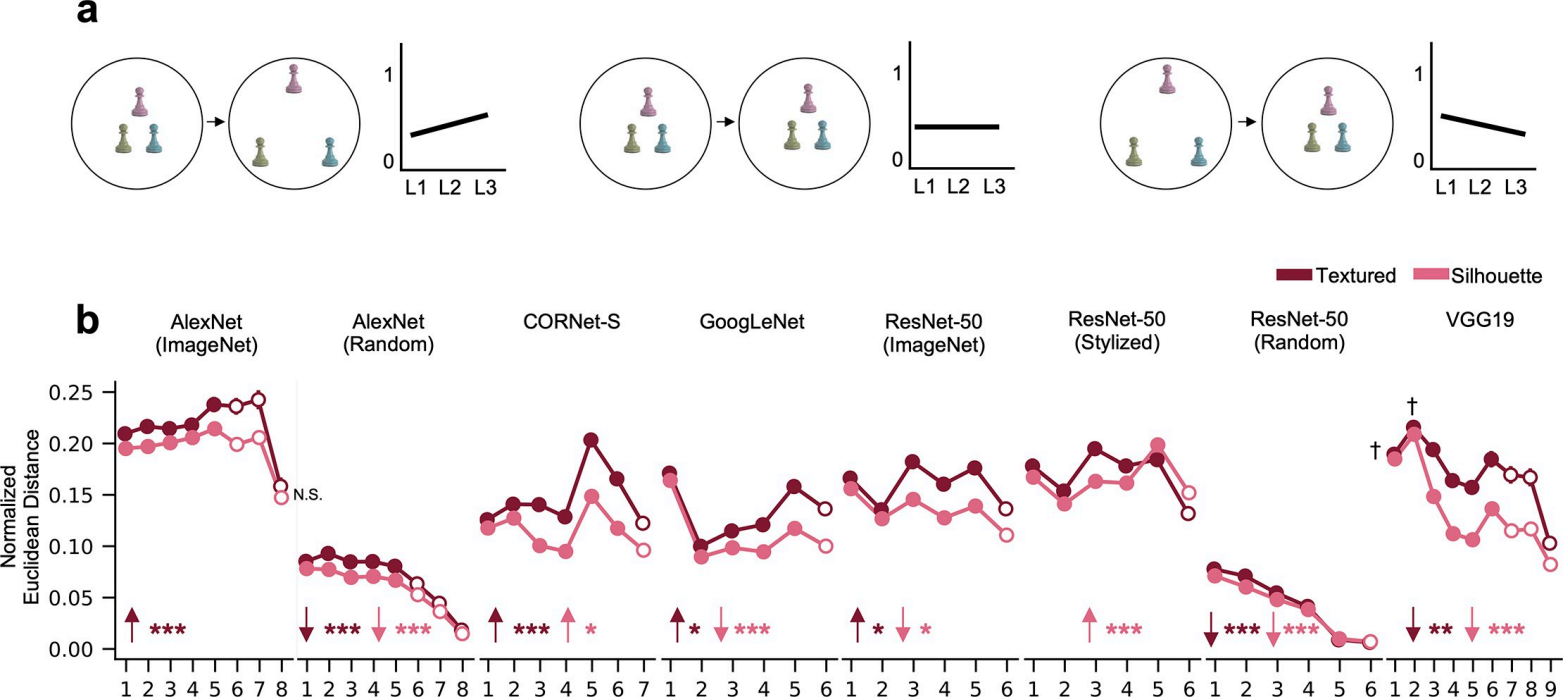
Magnitude of within-object color coding within each CNN layer. a. Schematic of three possible scenarios. In each scenario, the left figure illustrates the change in color space across two hypothetical CNN layers, with the distances among the same object in different colors reflecting the strength of color coding (with weaker and stronger coding corresponding to closer and more far apart arrangements, respectively). The right figure in each scenario illustrates how color coding strength may change across layers. Over the course of processing, color coding within an object can either grow more distinct (left panel), remain equally distinct (middle panel), or grow less distinct (right panel). b. Mean within-object color distances for each object. Results for the random networks are averaged across 100 random initializations. Fully-connected layers are marked by hollow circles and other types of layers sampled are marked by solid circles. Regression analyses were conducted to test for an aggregate increase or decrease in color representation over the course of processing; upward- and downward-facing arrows denote a significant increase or decrease respectively, with the level of significance denoted by the adjacent asterisks. Pairwise comparisons between the textured and silhouette stimuli were conducted for every layer; since a significant difference was found in every layer but a few, only the non-significant or trending layers are denoted. Different networks exhibit heterogeneity in how the strength of color coding varies across processing; however, the textured stimuli generally exhibit higher within-object color distances than the silhouette stimuli, and the untrained random networks *** *p* < .001, ** *p* < .01, * p < .05, † *p* < .1, N.S. = non-significant.

The previous analysis examined the extent to which the similarity structure among different colors varied across objects within each layer. However, another important characteristic of the representational geometry is the overall strength of color representation, i.e., the *magnitude* of the representational distances among the different colors within an object. The strength of color representation can increase (Fig 3A, left panel), stay the same (Fig 3B, middle panel), or decrease over the course of CNN visual processing (Fig 3C, right panel). While similarity structure and color representational strength capture different aspects of the representational geometry, such that representational strength can shrink while maintaining the same representational structure (as in Fig 3B), they are not entirely independent. For instance, a small shift in one of the representations would affect the color representational geometry much more when colors are close together and less distinctive than when they are far apart and more distinctive. This raises the question of whether the increasingly divergent color spaces of different objects observed over the course of CNN processing may have been a mere byproduct of a reduction in color representation in higher CNN layers. To address this, for each sampled CNN layer, we measured the mean between-color representational distance within an object, with this measure normalized by the number of units in each layer to enable between layer comparisons (see Methods for more details), and conducted several analyses to examine how these differences vary across conditions.

As shown in Fig 3, different networks exhibit different color representation profiles: while color representation increases across processing in AlexNet and then decreases in the final layer, it shows an overall decrease in VGG19, despite the fact that these networks contain similar layer types. Meanwhile, CORNet-S, GoogLeNet, and ResNet-50 do not show obvious patterns in how color representation changes across layers. The mean within-object color distance was significantly greater than zero in every condition and layer (one-sample t-tests, *ps* < .001). Both random networks (averaged across 100 random initializations) had the lowest color representation (with each layer being significantly lower than the corresponding trained layer; matched-pairs t-tests, *ps* < .001), which decreases further with processing, and the silhouette objects nearly always exhibited lower color representation than the textured objects (out of the 56 total cases, there were only three instances where non-significance or a trend was noted, see Fig 3; all others *ps* < .05, matched-pairs t-tests).

These results complement those of the previous analyses in several informative ways. First, the fact that including object textures increases the distances among the different colors of an object is another example of how form features, such as texture, can affect color representation in CNNs. Second, training the networks for object recognition increases the within-object color distances relative to the random networks, suggesting that training facilitates color information being retained rather than discarded. Third, importantly, these results suggest that the increasing divergence in color space geometry across objects that we observed in the previous analysis was *not* a byproduct of decreased color representation at higher CNN layers such that small perturbations would distort the color geometry to a greater degree. This is because even though color representational strength is lower in the random than the trained networks, color space geometry across objects is more similar in the former than the latter. A similar difference may be seen between the silhouette and the textured objects, with the former exhibiting lower color representation but higher color geometry across objects than the former. Lastly, even though AlexNet and VGG19 exhibit vastly different color representation profiles, both show increasingly different color geometry among different objects over the course of processing.

### Transformation of color space representations across CNN layers and architectures

**Fig 4:**
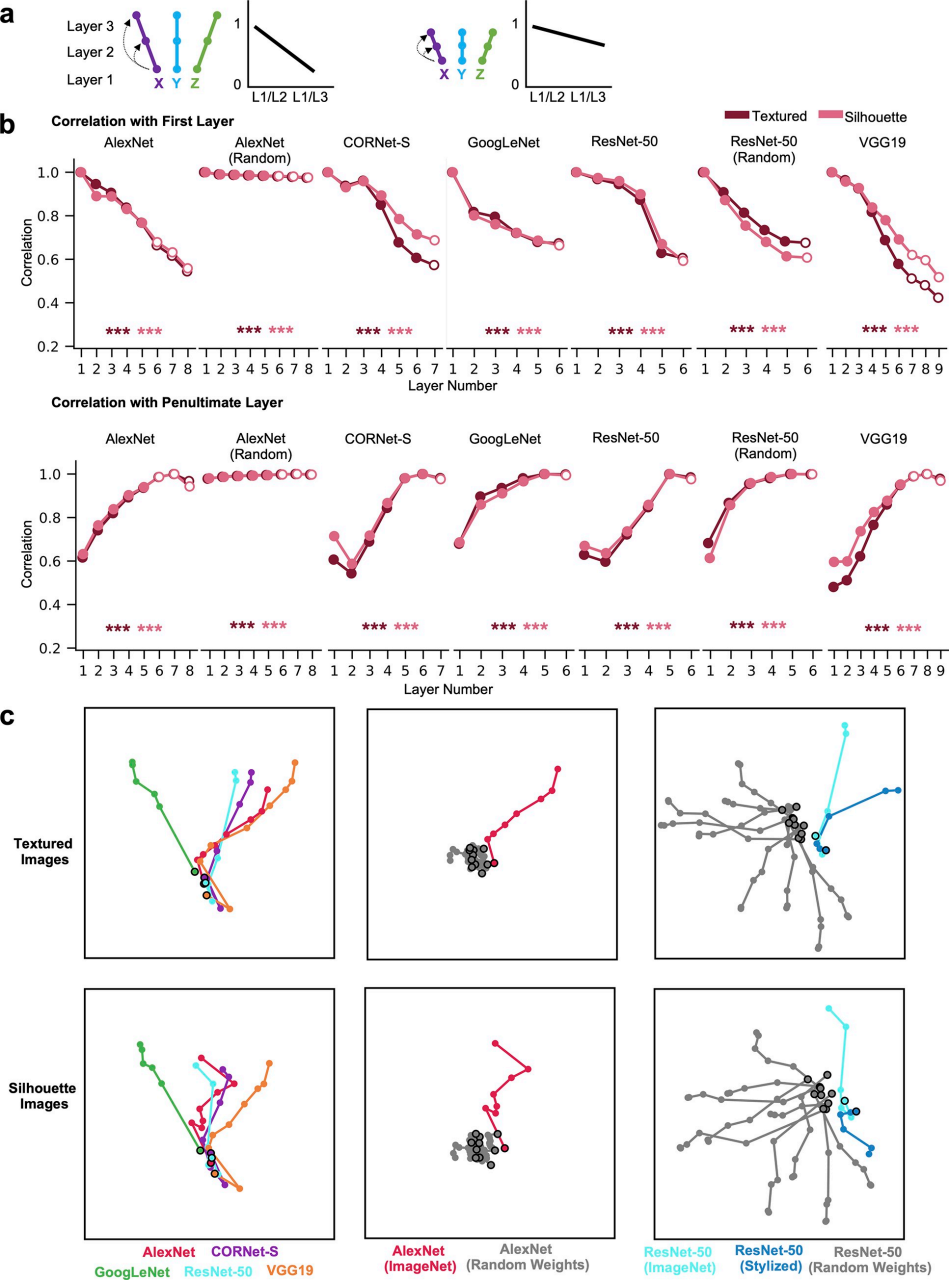
Color space representation across different CNN layers and different CNN architectures. a. A schematic illustration of two possible scenarios of color space correlation across layers within a CNN, using the same notations as those in Fig 2A. In this analysis, within each object, the color space structure from the first layer is correlated with each of the other layers, as shown on the left of each scenario. The averaged correlation over all objects for each layer is plotted in a line graph on the right of each scenario. In the first scenario, the color space structure within each object differs substantially across processing, resulting in a large decrease in correlation across layers. In the second scenario, the color space structure for each object remains relatively stable across processing, resulting in a relatively small decrease in correlation across layers. b. Mean within-object across-layer color space correlations for each network for the full set of 50 objects in the 12 colors calibrated in CIELUV color space for both the textured and silhouette versions of the objects. Top row shows the correlations with the first layer of each network, bottom row shows correlations with the penultimate layer of each network. Results for random networks are averaged across 100 random initializations of the network. Fully-connected layers are marked by hollow circles and other types of layers sampled are marked by solid circles. Linear regression was used to measure the downward or upward trend of the correlations for each object across layers. The resulting slopes were tested against zero for each of the two versions of the objects (with significance levels marked by maroon and pink asterisks, respectively). For all trained networks, the color space similarity within an object significantly decreases with more intervening layers, and correlations between early and late layers were fairly modest. This trend is far smaller for the versions of the networks with random weights c. MDS plots depicting color space correlation across different CNN layers and architectures. This was done by constructing a color space similarity matrix for each object, including its color space correlation across all sampled layers of all CNNs. The resulting correlation matrix was then averaged across objects and visualized using MDS. This was performed for the five trained networks (left column), AlexNet trained with ImageNet images and with 10 random-weight initializations (middle column), ResNet-50 trained with ImageNet images, trained with stylized ImageNet images, and with 10 random-weight initializations (right column), and for both the textured (top row) and silhouette images (bottom row). To facilitate comparison among models, within each of the two image sets the same MDS solution was computed across all models, but for visibility each respective subset is visualized in a separate panel. The black-outlined dots denote the first layer of each network. In the 5 trained CNNs (left column), color spaces are almost identical in the first layer and then gradually fan out during the course of processing, though in a similar overall direction. Color spaces in the untrained networks, however, differ substantially from the trained ones (middle and right columns). ** *p* < .01, *** *p* < .001.

In previous analyses, we examined differences in the color spaces across objects within a layer, and the magnitude of the within-object color differences within each layer. Here we took an orthogonal approach and tested how the color space of a given object may change across layers by correlating the color space vector of a given object between layers. Color coding for a given object may remain similar across layers, resulting in closely clustered color spaces across layers (Fig 4A, right), or it may transform substantially over the course of processing, leading to dispersed color spaces (Fig 4A, left). To quantify such transformations, for each object, we correlated the color space vector from each layer with the color space vector from the first and penultimate processing layers of the network (Fig 4B). We then used regression to examine whether the correlation significantly decreases with an increasing number of intervening layers from the reference (first or penultimate) layer. This was done both for the networks trained on ImageNet, 100 random initializations of AlexNet, and 100 random initializations of ResNet-50 (with correlation values averaged across random networks). Across the main set of 50 objects in 12 colors calibrated in CIELUV color space, in all cases, there was a significant and steady decrease in correlation with the target layer with increasing number of intervening layers (see the asterisks marking the significance level of the slope at the lower part of each plot). Even for the first few layers, although color space correlations within a layer were fairly high among the different objects (see Fig 2C), the color spaces of each object still differed *across* layers. For the random networks, while there was a statistically significant decrease in correlation with an increasing number of intervening layers, this effect was much smaller than in the trained networks, and in the case of the random version of AlexNet nearly nonexistent, suggesting that the color space transformations we observe are in large part induced by training the networks to recognize objects. Overall, color space was successively and substantially transformed over the course of processing for the trained networks, with the correlations between the color spaces at the beginning and end of processing being quite modest.

To understand how the color space of an object may be encoded differently among the different CNNs, for each of the 50 objects, we also correlated its color space vector across all CNNs and layers. We then visualized the resulting correlations, averaged over all objects, using MDS plots (after subtracting each correlation from 1 to convert similarities to dissimilarities). As shown in Fig 4C (leftmost column), across the 5 CNNs, for both the textured and silhouette objects, while the color spaces evolved substantially from their initial state over the course of processing (consistent with the quantitative analyses above), the color representations nonetheless evolved in a relatively similar way across networks, with the representations being almost identical in the first layer for all 5 CNNs and then gradually fanning out in a roughly similar direction during the course of processing, with GoogLeNet showing a greater divergence compared to the other CNNs. Of particular note, the penultimate layer of each network appears to encode color in a more similar manner compared to the penultimate layers of other networks than it does to the first layer of that network, suggesting that each network substantially transforms its color representations over the course of processing, but in a similar manner to other networks (with the exception of GoogLeNet). S3 Fig shows the exact between-network correlation values for both the initial and penultimate layers of each network, which in conjunction with Fig 4B corroborates the qualitative observations from the MDS plots regarding the color space differences among different models and layers.

To further understand how training on object classification may affect the color space of an object, we repeated the above analysis and correlated the color space vector of the same object across the layers of various instantiations of the same network with different training regimes: (1) across AlexNet trained with ImageNet images and 10 random initializations of AlexNet; and (2) across ResNet-50 trained with the original ImageNet images, ResNet-50 trained with stylized ImageNet images, and 10 random initializations of ResNet-50 (Fig 4C, middle and right columns respectively, and S4 and S5 Figs). In both cases, while color was initially encoded in a similar manner between the random and trained versions of the networks, over the course of processing, the color spaces for objects in the trained networks substantially diverged from those in the random networks. Interestingly, while the color spaces of the different random initializations of AlexNet tended to cluster together over the course of processing and did not diverge as the trained network did (consistent with the possibility that that the observed transformation in the color space was induced by training the network), those of the different random initializations of ResNet-50 diverged substantially but in different directions as those of the trained networks. On average, in the penultimate layers, color spaces for the two trained versions of ResNet-50 tended to be more correlated with each other than they are with the random initialization of the network; this was more so for the textured than the silhouette version of the objects. Additionally, the version of ResNet-50 trained on ImageNet was approximately as similar in its penultimate layer to the version of ResNet-50 trained on stylized ImageNet as it was to the other networks trained on ImageNet (S3 and S5 Figs), suggesting that both a network’s architecture and its training can contribute to the transformation of color information in a network.

Overall, these results demonstrate that, within a given network, color representations for each object transform dramatically over the course of processing. Across the different networks, color spaces evolved in a roughly similar manner across the trained networks. This transformation of color space was not a mere byproduct of a network’s intrinsic architecture, as the color spaces of untrained networks evolved substantially differently from the trained networks.

### Transformation of color space similarity across CNN layers

**Fig 5:**
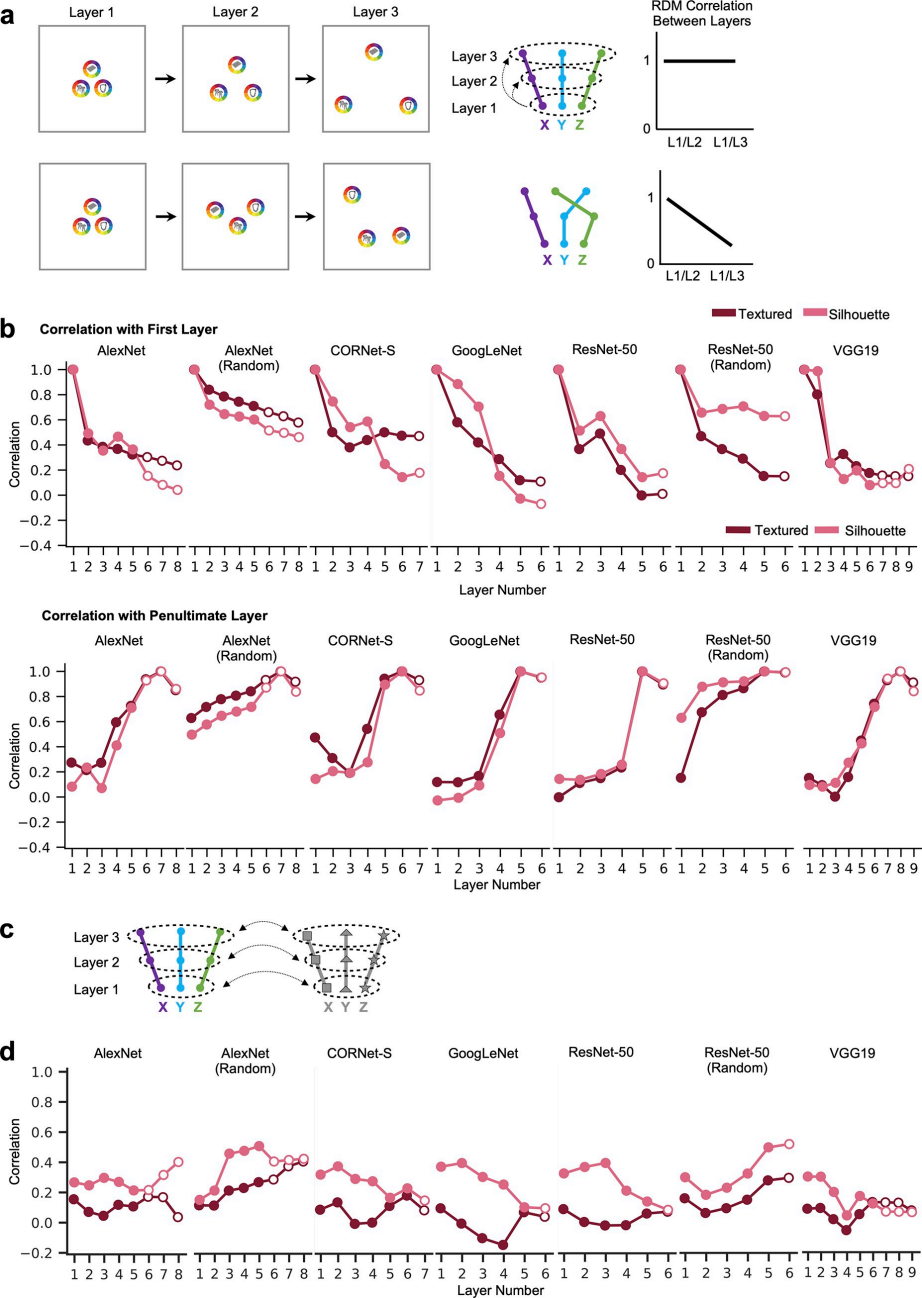
The evolution of color space similarity among objects across CNN layers and the dependence of color space similarity on object form similarity. a. A schematic illustration of two possible scenarios of the evolution of color space similarity among objects across CNN layers, using the same notations as those in Fig 2A. In this analysis, we examine whether or not patterns of color space similarity among objects (as shown on the left) are preserved across layers by correlating the color space similarity matrix (i.e., the second-order RSM quantifying the similarity among the color spaces of different objects) from the first layer with each of the other layers, as shown in the middle. These correlations are then plotted in a line graph on the right. In the first scenario (top row), the relative color space similarity among the different objects is preserved in the different CNN layers (i.e., the configuration of the three color spaces stays the same across the different layers), even as the absolute similarity among color spaces decreases. In the second scenario, the relative color space similarity is not preserved in different CNN layers (i.e., the configuration changes across the different layers). b. The correlations of the color space similarity across different CNN layers for the full set of 50 objects in the 12 colors calibrated in CIELUV color space for both versions of the objects. Top row shows the correlations with the first layer of each network, bottom row shows correlations with the penultimate layer of each network. Fully-connected layers are marked by hollow circles and other types of layers sampled are marked by solid circles. In most cases, correlations between the early and late layers are fairly modest. Results for random networks are averaged across 100 random initializations of the network. c. A schematic illustration of comparing color space similarity and object form similarity, using the same notations as those in Fig 2A. In this analysis, the achromatic object form similarity matrix is extracted for each CNN layer and then correlated with the corresponding color space similarity matrix of that layer. d. Correlations between the form similarity and color space similarity for each layer of each network for the full set of 50 objects in the 12 colors calibrated in CIELUV color space for both versions of the objects. Results for random networks are averaged across 100 random initializations. No reliable trends were evident, but in general correlations were modest.

To understand how color space similarity may change across objects over different CNN layers, instead of testing the color space of a single object, here we asked: is the profile of color space similarities among different objects preserved across processing (Fig 5A top), or does the pattern of color space similarities among objects change throughout processing (Fig 5A bottom)?

To test this, for the main set of 50 objects in the 12 colors calibrated in CIELUV color space, for each CNN, we took either the first or penultimate layer as our target layer and first generated its color space similarity RSM by performing all pairwise correlations of color space vectors between objects. We then vectorized the off-diagonal elements of this RSM to form a “color space similarity” vector and correlated this vector with those from all other layers of the CNN. This was done for both the networks trained on ImageNet, and on 100 random initializations of AlexNet and ResNet-50 with untrained weights. We found that for all networks, correlations decreased as we moved away from the reference layer, with the first and last layers being only moderately correlated (Fig 5B). This transformation occurred in the untrained networks as well, though to a lesser extent than in the trained networks. Thus, if two objects had a highly similar color space at the beginning of a CNN, they did not necessarily have a highly similar color space at the end of the CNN. Patterns of color space differences among objects appear to dramatically change throughout the course of processing.

### The effect of object form similarity on color space similarity

It is possible that color space similarity covaries with object form similarity, such that a small change in form features leads to a small change in the associated representational geometry for color. However, given that each feature can vary relatively independently of the other feature, it is also possible that color coding does not closely follow form coding. To arbitrate between these two possibilities, and better understand what factors might be driving the divergence in color space across objects, for the main set of 50 objects in the 12 colors calibrated in CIELUV color space, for each CNN layer, we first performed all possible pairwise correlations of the CNN layer output for the grayscale versions of the object forms to form an object form similarity RSM and vectorized the off-diagonal elements of this RSM to form an object form similarity vector. We then correlated this object form similarity vector with the color space similarity vector from the same CNN layer (Fig 5C). If similar object forms had similar color space structure, we expected to obtain a high correlation between the two. We performed the analysis for both the networks trained on ImageNet, and 100 random initializations of AlexNet and ResNet-50. As shown in Fig 5D, correlations varied across layers and networks, showing no consistent pattern; correlations tended to be higher for the silhouette than for the textured stimuli, but tended to be modest. Interestingly correlations existed for the random networks as well, suggesting that a network’s intrinsic architecture can induce a correlation between the shape similarities of different objects and the color spaces similarities of those objects. Overall, color space similarity does not closely track object form similarity, suggesting some separation between the two, and that the difference in color space similarity between two objects does not scale linearly with the difference in their form features.

## Discussion

Despite decades of neuroscience research, we still lack a full understanding on how feature conjunctions are represented in the primate brain. In this study, we took advantage of the recent development in CNNs trained to perform object classification and examined how such an information processing system jointly represents different object features across the entire processing hierarchy of a CNN. We did this through using a variation of RSA to examine how color coding varies across different objects, which provides an index that reflects the extent to which color and form are encoded in an interactive, as opposed to independent, manner. Our investigation not only allowed us to gain insight into the internal representations of CNNs, but also enabled us to develop a novel network-based stimulus-rich approach to study feature binding across the entire network and a large stimulus set, which can be easily implemented to study feature binding in biological visual systems. Although we tested the joint coding of color and form here, our approach can be applied to study the joint coding of any pair of features: the variation in coding for feature X across values of feature Y can be computed by first computing the similarity space for feature X *within* each value of feature Y, then correlating these similarity spaces *across* each value of feature Y.

With this approach, we found that color coding increasingly varies across different real-world objects in higher levels of each CNN. This held true for both the naturally textured stimuli and the uniformly colored “silhouette” stimuli, suggesting that interactive coding of color and form in higher CNN layers exists for global form features alone (which are preserved in the silhouettes), even in the absence of texture and much of the category-diagnostic visual information. The textured interior of an object form, however, did further increase the amount of interactive coding between colors and forms, likely due to the presence of additional form features in these textured objects. This interactive coding appears to be quite general, as it was present not only for complex real-world object forms, but also for minimally simple oriented bar features with stimulus size equated. Moreover, this observed interactive coding was not a mere byproduct of a decrease in color representation throughout processing, since in various cases an increase in interactive coding was accompanied by *increased* color representation, contrary to what this alternative explanation would predict. Finally, this effect did not depend on the distance metric that was used to compute the representational geometry of the features, nor did it depend on our particular selection of color space used to calibrate the luminance and saturation of the images.

All sampled networks contained both convolutional and fully connected layers, including the final category output layer. The increasing degree of interactive coding we observed throughout processing was found both in the fully connected layers prior to the category readout layer, and the category readout layer itself. In particular, the most clear decrease in color space correlation did not suddenly appear in the fully connected layer, but rather it starts at least one sampled layer earlier, sometimes several sampled layers earlier as in AlexNet, CORNet-S and VGG19, and then continues smoothly to the fully-connected layer(s). A priori, one would assume that the final fully connected layer encodes object category orthogonally to color, since it is trained to output category labels. However, prior work has shown that fully connected layers encode not just information about object category membership, but also information about features such as shape, position, spatial frequency, and size [19, 36]. The present results further show that there is both a significant amount of color representation and a greater amount of color and form interaction in the final compared to the first sampled layer, with the amount of interaction steadily increasing during the course of visual processing. Additionally, while the networks we examine vary broadly with respect to parameters such as the number of units and layers, we observe the same increasing entangling of color and form information as processing proceeds in each network. This suggests that it is a truly general property of CNN information processing rather than a quirk arising from the architecture of any particular network or layer type.

It is important to note that in the input to CNNs (that is, a grid of RGB pixels) color and form are initially encoded “independently”, in that color is present in the values of the RGB values within each pixel, and “form” is implicitly present in the exact spatial configuration of pixels. Consistent with this independence, in all the networks examined, mean global correlation of the color similarity matrices across objects in the first layer is very close to 1 (r > .98 for all networks, r > .99 for every network except AlexNet). Thus, our correlation measure is capable of reflecting independent coding of color and form when it is present in the network. The decline of this correlation over the course of visual information processing in CNNs can only indicate a loss of independent coding and an increase in interaction.

Several studies have shown that even random networks can exhibit interesting tuning properties, such as tuning to numerosity [41], or predictiveness of visual responses in mouse cortex [42]. Here we found that the interaction between color and form coding was not a mere byproduct of the intrinsic architecture of a CNN, as the interaction effect was profoundly attenuated in untrained CNNs with random weights. That said, the increasing color-form interaction over the course of processing was still significant for the random networks, suggesting that a small part of the effect arises from intrinsic aspects of the CNN’s architecture. The interaction between color and form coding did not appear to depend on the existence of natural covariation between form and color in the training set, as the magnitude of interactive tuning is just as large (and in the case of the textured objects, larger) in a CNN trained on objects stripped of their naturalistic form-color pairings. Thus training for object recognition is needed to produce the interactive coding of color and form, even when no consistent color and form pairing is present during training. This suggests that the CNN architecture alone is not sufficient to give rise to the interactive form-color coding we observe (the weights must also be tuned to appropriate values), and that object recognition training automatically gives rise to increasingly tangled color and form representations in higher levels of processing, even when color is not informative to object recognition after training.

To our knowledge, only one study has examined the joint encoding of color and form features in CNNs: [25] examined the responses of several individual color-selective CNN units in both the first and last convolutional layer and found that they were also sensitive to both the color and the orientation of an image patch. Based on this result they suggest that color and form are “entangled” at all stages of processing. At first glance, this seems to conflict somewhat with our finding that color and form are first encoded orthogonally but are increasingly encoded interactively with further processing. However, this difference can be readily explained by the difference in analysis approach. These authors examine interactive color-form tuning in single units, whereas our method involves examining changes in the population-level representational geometry of color coding across form changes. Interactive color-form coding in single units could coexist with a stable population-level representational geometry for color coding across forms if color and form interact in the same way across different colors. For example, this would occur if for every unit selective for vertical red-green edges there exists a unit selective for vertical blue-yellow edges. This distinction highlights an important point in studying neural networks whether biological or artificial: the way that a single unit jointly encodes two features does not transparently reveal how these two features are jointly encoded at a population level.

[25] also reported that non‐color selective CNN units have a range of spatial frequency (SF) preferences, whereas color selective units largely prefer low SF. This result suggests that spatial frequency may play a role in modulating the amount of color and form interaction, with the amount of interaction differing at different SF. Consequently, if we equalize SF among the different objects, we should no longer see interactive coding of color and objects. However, even for a stimulus set containing identical rectangular bars with different orientations (and thus the same SF profile), the interactive coding between color and object still increases over the course of CNN processing (see Fig 2D). Thus, while it is possible that SF differences may further contribute to interactive coding, a topic that warrants further detailed investigation, there nevertheless exists a genuine effect of increasing color and form interaction in CNN processing that is independent of the SF content of the objects.

In additional analyses, we found that an object’s color space greatly diverged from its initial color space over the course of processing and that two objects with a similar color space at the beginning of processing did not necessarily have a similar color space at the end of processing. Thus the color space representation for a given object as well as the relative similarity of color spaces between objects dynamically changed over the course of processing. These color space transformations were greatly reduced in the untrained networks, suggesting that training networks for object recognition induces a transformation in how color is encoded over processing. Moreover, the color space of an object tended to transform in similar ways across the trained networks, but differently in the untrained networks. This relative consistency across the trained, but not the untrained, networks with vastly varying architectures suggests that this resculpting of color space may be of adaptive value for the network’s object classification task. Interestingly, the achromatic form similarity of two objects only weakly predicted the similarity of their respective color spaces. This demonstrates that, in general, color space similarity does not closely track object form similarity, suggesting some separation between the two. The untrained networks also exhibited a correlation between shape similarity and color space similarity, suggesting that these correlations may be a byproduct of a network’s intrinsic architecture.

Overall, these results show that colors are not represented similarly across different objects in an orthogonal manner in CNNs. Rather, colors are encoded increasingly differently across objects in an interactive and object-specific manner during the course of CNN processing. This is more consistent with a late feature integration account (but without needing an additional binding operation), rather than color and form being represented in an initially entangled and intermingled fashion and only being represented separately and explicitly in later layers. To our knowledge, these results provide the first detailed and comprehensive documentation of how color and form may be jointly coded in CNNs, unveiling important details regarding the algorithms employed by CNN for visual processing, which up to now have remained largely hidden. While some studies have examined the coding of complex, high-level form features in CNNs, such as those involved in face recognition [43], this study is the first to document the joint population coding of two completely *different* visual features, color and form. Moreover, the present study examines this interaction both for complex, naturalistic form features and simple form features, in the case of the oriented bar stimuli.

It should be noted that interactive tuning does not imply that there exist units tuned exclusively to a single color/form conjunction (a “grandmother unit”); units could plausibly be tuned to heterogeneous combinations of color and form combinations in a “mixed selectivity” coding scheme. Such a coding scheme has been reported in the macaque prefrontal cortex for the coding of stimulus identity and task and has been shown to vastly increase the neural representational capacity of that brain region [44]. The present results suggest that an interactive coding scheme along these lines may be more prevalent and can automatically emerge in a complex information processing system even though, compared to a biological brain, CNNs have a relatively simple structure, consisting only of a single feed-forward sweep and lacking mechanisms such as feedback connections (except for the recurrent network, CORNet-S, we included here) and oscillatory synchrony. Such a coding scheme may well be used by sensory regions in the primate brain to support the flexible encoding of a wide range of sensory feature combinations. Indeed, although initial evidence from visual search [3] and neuropsychology studies [45] suggests that color and form might be initially encoded independently, and only combined in a late binding operation, other strands of evidence suggest that color and form might be encoded in an interactive manner early on during processing [46]. For example [29], and our own work [47] have shown that nonlinear tuning for color/orientation combinations might emerge as early as V1, V2, V3, and V4. Consistent with the present observation, interactive coding of color and form has also recently been observed in the color selective neurons of macaque color patches [28].

Despite its significance in visual cognition, how feature conjunctions are coded in the human brain remains unresolved. A population code that instantiates interactive tuning for feature combinations, as we observe here, is a candidate mechanism that should be explored in more detail, and analogous analyses should be applied in monkey neurophysiology and human fMRI studies to see if similar response profiles exist in the primate brain. Indeed, given that a number of previous studies have shown that the representations formed in lower CNN layers better correlate with lower than higher primate ventral visual regions and, conversely, the representations formed in higher CNN layers better correlate with higher than lower primate ventral visual regions [10–16], our results may be used to directly predict and compare with responses from corresponding primate cortical regions.

To summarize, despite the success of CNNs in object recognition tasks, presently we know very little about how visual information is processed in these systems. The present study provides the first detailed and comprehensive documentation of how color and form may be jointly coded in CNNs. Our development of a novel network-based stimulus-rich approach to study feature binding in CNNs can be easily implemented to study neural mechanisms supporting feature binding in the primate brain. Equally importantly, the discovery of the interactive coding scheme used by CNNs to encode feature conjunctions could be a viable coding scheme that the primate brain may employ to solve the binding problem.

## Methods

### CNN selection

We chose five CNNs in our analyses: AlexNet, CORNet-S, GoogLeNet, ResNet-50, and VGG19. These CNNs were selected based on several different criteria. AlexNet [31] was included for its relative simplicity, and prevalence in the literature. VGG19 [32], GoogLeNet [33], and ResNet-50 [34] were chosen based on their high object recognition performance and architectural diversity. Additionally, both AlexNet and VGG19 have a shallower network structure, whereas GoogLeNet and ResNet-50 have a deeper network structure. Finally, CORNet-S [35], a shallow recurrent CNN designed to approximate the structure of the primate ventral visual pathway, was included for its high correlation with neural and behavioral metrics. This CNN has recently been argued to be the current best model of the primate ventral visual regions [35]. For most analyses, we used pre-trained implementations of these CNNs optimized for object recognition using ImageNet [30]. The exact training method differed somewhat among networks; specifically, AlexNet, VGG19 and ResNet-50 use cropping, horizontal flips, and RGB adjustments (simulating changes in lighting) for data augmentation, whereas CORNet-S only uses cropping and horizontal flips, and GoogLeNet only employs cropping. To understand how the specific training images would impact CNN representations, we also examined responses from an alternative version of ResNet-50 that was trained on stylized ImageNet images in which the original texture of every single image was replaced with the style of a randomly chosen painting. This biased the model towards representing holistic form information rather than texture information [40]. Finally, in order to determine to what extent the architectural parameters of a network (number of layers, kernel size, etc.), independent of any training, affects the results, we also examined multiple initializations of AlexNet and ResNet-50 with randomly assigned weights and no training. The PyTorch implementations of all models were used, and custom scripts designed to interface with PyTorch were used for all analyses [48].

Since these CNNs have varying, and often large, numbers of layers, we performed analyses over a subset of 6 to 9 layers in each model, in order to simplify analysis and roughly equate the number of layers analyzed in each model. The first layer, several intermediate layers, the penultimate layer (i.e., the last layer before the object category label outputs), the final layer (i.e., the object category label output layer or the classification layer), and any other fully-connected layers were used for each model. Selection of intermediate layers varied based on the model, but since all CNNs we examined tended to be structured into architecturally significant “segments” (e.g., VGG19 has repeated “segments” composed of alternating conv and relu layers followed by a pooling layer, CORNet-S has “segments” meant to correspond to different visual brain areas etc.), we included the intermediate layer corresponding to the boundaries between “segments”. These intermediate layers were also chosen in such a manner as to sample the network as evenly as possible, and to at least roughly equate the number of layers extracted from each network. These criteria, applied uniformly across networks, sometimes resulted in different layer types being sampled in different networks. For example, convolutional layers were used in AlexNet because there are five convolutional layers roughly “evenly spaced” throughout the network (as opposed to the pooling layers, of which there are only three, with a much bigger gap between the second and third than between the first and second), whereas pooling layers were used in VGG19 because there are five roughly evenly spaced pooling layers, each of which separates a “chunk” of multiple convolutional layers in a row. Our layer selection thus captures meaningful transitions in the processing hierarchy of each network. In a control analysis, we further showed that our sampled layers capture the overall processing trajectory of the network and that the trajectory does not change with the types of layers sampled, as long as they are adjacent to each other in the processing pipeline (S1 Fig; details of this analysis is described later in Methods). Although fully connected layers (including the classification layer) differ from early layers in the network in that they do not follow a weight-sharing constraint over space, past work has found that they encode not just information about object category membership, but also information about features such as shape, position, spatial frequency, and size [19, 36], making it appropriate to examine how they jointly encode the features of shape and color at the end of CNN information processing. The specific layers we included (with their PyTorch labels given) are listed in Table 1. Throughout the text, we adopt the convention of labeling layers by the kind of layer, followed by the number of times that kind of layer was used up to that point in processing (e.g., the third convolutional layer is conv3).

In cases where we wished to compare coding over the course of CNN processing or comparing coding at the beginning and end of processing in the network, the first through *penultimate* layers, or the first and *penultimate* layers, were used, respectively. Although the final classification layer contains feature representation as stated earlier, given that this layer is constructed to pool the processed object information together to assign category labels rather than to further process object information, choosing the penultimate layer here would better reflect the state of visual information representation at the end of CNN processing.

In order to compare our results across different CNNs, for some analyses we coded a variable, layer_fraction, that reflects what fraction of a network’s layers have been traversed up to a given layer in the course of a CNN’s processing hierarchy (all layer types were included). For example, the first layer in a ten-layer network would have a value of .1 for this variable, and the final layer would have a value of 1.0.

### Stimulus selection

We used a set of real-world object stimuli from Brady et al. (2013) as our main object stimuli. This stimulus set consists of images (400 x 400 jpegs) of 540 different objects, where the colored portions of these objects are all initially of the same hue (so as to facilitate manipulating the colors of the objects in a consistent way). To derive the stimuli used in our analyses, we selected a smaller subset of these objects (as detailed below), and then manipulated the color and texture of these objects.

#### Main object stimuli in CIELUV colorspace

For our main stimulus set, we chose 50 objects intended to be maximally dissimilar with respect to their high-level visual features. To do this, we converted the initial 540 objects to grayscale, ran them through AlexNet, and extracted their activations from AlexNet’s penultimate (last pre-classification) layer, FC2. We then constructed a representational similarity matrix (RSM) by computing all pairwise correlations of the CNN output from layer FC2 for each object with each other, and used this RSM to select a set of 50 objects whose mean pairwise correlation was minimally low. With this procedure, the mean pairwise similarity went from *r* = .25 (min *r* = -.02, max *r* = .92) for the original set of 540 objects, to a mean pairwise similarity of *r* = .13 (min *r* = -.02, max *r* = .78). The resulting set of 50 objects are shown in Fig 1A; visual inspection confirms that the resulting object set spans a wide range of different form and internal texture features.

We then recolorized each of the 50 objects, roughly following the procedure outlined in [28]. This procedure guaranteed that all stimuli had the same mean luminance and saturation over the non-background portions of the image. Equating mean luminance was necessary in order to equate each image’s contrast with the background, and ensure that any results were not mere byproducts of contrast-sensitive mechanisms rather than color processing as such. Clearly, even a color-blind network would encode lingering contrast differences, so equating contrast across colors is necessary to ensure that any results are genuinely driven by color-sensitive mechanisms. Equating mean saturation was necessary to ensure the validity of some of our analyses; in particular, since we examine whether color coding varies based on form, failing to equate saturation could introduce spurious results, since a relatively unsaturated object image would by definition have less hue variation (this is especially problematic since the RGB values fed into the network are integers, so reducing the hue variation could hide important structure by introducing a loss of precision due to rounding). Additionally, without equating saturation, it would remain a possibility that variability in certain units could be driven by the degree of saturation, rather than hue per se. Since luminance and saturation are not explicitly encoded in the RGB color space, we adopted the approach of first tuning the hue, saturation, and luminance of every image (every color of every object) in LUV color space before converting the image back to RGB to feed into the network. Each object stimulus was colored in each of 12 different hues, evenly spaced around the colorwheel. Specifically, we converted all images to the CIELUV colorspace, which is constructed such that equal distances in the space correspond to roughly equal psychophysical differences; this was done so that we could use the same stimuli on a future study comparing our results to those of human observers. Next, we computed the mean luminance (L) of each stimulus over the non-background portion of each stimulus, and did the same for the saturation (computed as $\sqrt{u^{2}+v^{2}}$, where u and v are the two chromaticity coordinates in the LUV color space). For each image, a constant value was then added to the luminance and saturation of the non-background pixels so as to bring the mean luminance and saturation of that image to target values that were equated across all objects. This procedure sometimes resulted in pixels whose values overflowed past the range of permissible LUV luminance and saturation values; in cases where this occurred, the variance of the luminance or saturation about the mean was shrunk until all pixel values fell within the permissible boundaries. Once the luminance and saturation for each pixel were set in this manner, each object was colored in 12 different hues by rotating the U and V (hue) coordinates in each pixel to 12 equally spaced angles. Additionally, a grayscale version of each stimulus was created by setting U and V to zero, while keeping the luminance channel the same. This procedure preserves relative saturation and luminance patterns that were present across each original image, while manipulating hue and equating mean saturation and luminance. The target mean saturation and luminance were derived through successive adjustments, until applying the above transformations to each image did not take any pixel’s LUV values outside of their allowable range. Mean saturation was required to be relatively low, due to the nonlinearities of the LUV color space; specifically, a high saturation value may be possible for one luminance/hue combination, but not others, so saturation had to be kept within relatively narrow boundaries. Stimuli were converted from LUV back to RGB color space prior to being run through the networks, as the networks were trained on RGB images. Since internal texture is preserved for these stimuli, we henceforth refer to them as the “Textured” stimuli.

In addition to the above method, which preserved the internal texture of the stimuli, we also constructed a version of each stimulus that consisted of a uniformly-colored “silhouette” of the image (henceforth “silhouette” stimuli), thereby removing internal texture information while sparing global form information. These were constructed by replacing every non-white pixel in the object with a pixel of a uniform color. Twelve such silhouette images were created from each object, using the same 12 hues and the same mean luminance and saturation values as were used for the Textured stimuli. We performed this manipulation because some evidence suggests that CNNs may prioritize texture over global form features [39], so employing silhouette stimuli allowed us to examine whether our results hold in the absence of texture. Example stimuli from these two methods, in the 12 possible colors, are shown in Fig 1B.

#### Main object stimuli in synthetic HSV colorspace

The CIELUV color space, while widely used, implements a specific parametrization of luminance and saturation that is based on human psychophysical judgments, which may not be applicable to how CNNs represent visual information. To ensure that our results do not depend on these idiosyncrasies of the CIELUV color space, we constructed a stimulus set whose colors were calibrated in a novel color space that we designed not to align with human data, but with the RGB input that CNNs receive. Specifically, we used a variation of the common hue/saturation/luminance parametrization calculated over RGB values, which we call Synthetic HSV. Some such parametrizations require taking maxima and minima of the RGB channels, an operation which is not available to convolutional kernels. Thus, luminance was stipulated to be the mean of R, G, and B; this definition assigns equal weights to the three channels (unlike some HSV parametrizations, which weight the three channels differently to account for human psychophysical performance), and uses an operation (taking the mean) that is easily implemented by convolutional kernels. Intuitively, saturation reflects the dissimilarity of a color from neutral grey; thus, saturation was stipulated to be the Euclidean distance in RGB coordinates from a color to neutral grey of the same luminance. Once these values are fixed, the range of possible RGB values forms a circle in the 3D RGB space. This occurs because restricting the RGB values to have a fixed average—and therefore a fixed sum—constrains the range of possible RGB values to fall on a single plane, and further restricting the RGB values to have a fixed Euclidean distance from the neutral gray point on that plane (where R = G = B) selects a circle of RGB values on that plane. Within this circle, we stipulated that the RGB triplet with the highest R channel corresponds to a hue value of 0°. To keep the overall level of luminance and saturation in a similar range to the stimuli calibrated in the CIELUV color space, we set the mean luminance and saturation (in Synthetic HSV) for these stimuli to the values of these parameters for one of the colors calibrated in CIELUV space (specifically, the one with the highest red channel in RGB space). We then constructed stimuli with these chosen values as their mean luminance and saturation, with 12 hue values evenly spaced around 360°. After calibrating the stimuli in synthetic HSV, stimuli were converted back into RGB to feed into each network. Stimulus construction was otherwise identical to the procedure for the stimuli colored based on CIELUV.

#### Oriented bars in CIELUV colorspace

To examine the extent to which our results may hold for stimuli equated in their spatial coverage and for simpler stimuli than the naturalistic object stimuli that were used in the study, we constructed a set of oriented bar stimuli (Fig 1C). Twelve orientations, ranging in even increments from 0° to 180°, were used, and each was uniformly colored in the same twelve isoluminant and isosaturated colors that were used for the object stimuli (using CIELUV space).

### Analysis methods

For all analyses, images were fed into each network. Next, unit activations were extracted from each sampled layer and flattened into 1D vectors in cases where the layer was 3D (e.g., if it was a convolutional layer).

#### Visualizing color space representation across objects and CNN layers

We used representational similarity analysis (RSA) to measure conjunctive tuning for color and object form. Specifically, we examined the extent to which the representational structure for color changes across the different object forms. To the extent that the representational structure of color varies across the object form, it would provide evidence that CNNs encode color and object form not independently, but interactively.

As an initial analysis, we visualized how the color spaces for two example objects differ at the beginning and end of a CNN. Specifically, we extracted the patterns for all 12 colors of two example objects from the first and the penultimate layers of AlexNet (which are Conv1 and FC2). Within each layer, we performed all pairwise Pearson correlation among the 12 patterns for each object to create a representational similarity matrix (RSM, with the value for each cell being the Pearson correlation coefficient); each value was then subtracted from 1 to convert it to a dissimilarity matrix. Using multidimensional scaling (MDS), we visualized the resulting dissimilarity space (Fig 1D).

Next, we visualized how the color spaces of six representative objects might diverge over the course of processing in AlexNet (Fig 1E). To do this, for each object and for each sampled layer of AlexNet, we first constructed a “color space” RSM by performing all pairwise Pearson correlations of the patterns associated with the 12 different colors of that object (with the value for each cell of the matrix being the Pearson correlation coefficient). We vectorized the off-diagonal value of this RSM to create a “color space” vector. Next, we performed all pairwise correlations of these “color space” vectors across objects and layers to form a “color space similarity” RSM that quantifies how similarly color is coded in different objects and layers. After converting the matrix to a dissimilarity matrix by subtracting each value from 1, we then used 2D MDS to visualize the similarity of the different color spaces across different objects and CNN layers. Fig 1F shows the full color space *similarity* matrix among all 50 objects for Conv1 and FC2 of both the trained and untrained version of AlexNet.

As an additional analysis to examine whether the results depend on the exact layer type selected (S1 Fig), we performed the same visualization for three objects, but sampling from *all* layers of AlexNet. We found that our sampled layers capture the overall processing trajectory of the network and that the trajectory does not change with the types of layers sampled, as long as they are adjacent to each other in the processing pipeline (S1 Fig).

Following these qualitative observations, to provide a comprehensive and quantitative description of color representation across different objects and CNN layers, we performed a series of analyses. Specifically, we quantified (1) within each layer, how the color similarity structure varies across objects (Fig 2A), (2) how strongly color is represented within objects (Fig 3A) (3) within each object, how color is coded across different layers of a CNN and different CNNs (Fig 4A), and (4) whether or not color space similarity among the different objects within one layer is preserved across CNN layers (Fig 5A). We also quantified how color space similarity between two objects may be determined by their form similarity at a given CNN layer (Fig 5C). These four analyses are described in detail below. All the analyses were performed for both the Textured and Silhouette stimuli.

#### Quantifying color space differences across objects within a CNN layer

To understand how color is coded across objects in each CNN layer, we first created a “color space” vector for each object in each layer of each CNN as described above for our main stimulus set of 50 objects and 12 colors calibrated in CIELUV color space. We then performed all pairwise correlations of these “color space” vectors for all the objects for a given CNN layer. We next averaged these correlation values within each layer and used a line plot to visualize how the mean colorspace correlation changes over layers of a given CNN (Fig 2). Fig 2B shows the color space correlations between every pair of objects in each layer of AlexNet, for the colored object stimuli calibrated in CIELUV color space. Fig 2C shows the mean pairwise color space correlations among every pair of objects for the five trained networks we examined. To assess the statistical significance of any change across layers, for each pair of objects, the correlation values between the color spaces of those two objects from the first to the penultimate layers were regressed onto the CNN layer positions (using the layer_fraction variable described in the CNN Selection section). The resulting slope from this regression reflects the degree to which the color space similarity for these two objects increases or decreases over the course of the network. A positive slope would mean that the color spaces for these two object forms become progressively more similar over the course of processing. A one-sample t-test was used to test the slopes from all possible object form pairs against zero to assess whether the average slope was significantly different from zero (that is, whether the mean color space similarity between objects tends to change over the course of processing). Additionally, a matched-pairs t-test was used to assess whether the slopes significantly differed between the textured and silhouette images. Note that these regression analyses do not require a strictly monotonic decrease in color space correlations as processing proceeds, but only whether there tends to be an increase or decrease on average.

To examine whether our effects hold not only for complex, real-world objects but also for simple artificial forms, we repeated the same analysis on simple oriented bar stimuli, where the different object “forms” were simply different orientations of a centrally placed bar stimulus (Fig 2D). This allowed us to examine whether the results would hold for stimuli equated in their overall spatial coverage and whether results obtained from complex nature objects hold for simple form stimuli.

We also performed the same analysis in a number of control conditions, to examine whether specific choices regarding the stimulus set and analysis method affect the results. As our first control, to test how the particular similarity measure we used may impact the results, we repeated our analysis, but used Euclidean distance as our initial similarity metric instead of Pearson correlation (**S2A Fig**); this was done because Euclidean distance, unlike Pearson correlation, is an unbounded metric, and we sought to ensure that the choice of the specific similarity measure did not affect the results (Pearson correlation was still used as the second-order similarity metric to quantify the differences *between* the color spaces of different objects). As our second control, to examine whether our results depended on our particular choice of color space, we repeated the same analysis on the same set of objects whose colors were calibrated in the synthetic HSV space described above, instead of the LUV space used in our main stimulus set (**S2B Fig**).

#### The effect of training on CNN color space representation

In order to assess whether the naturally occurring consistent color and form conjunctions present in the training images were necessary to produce the results we observed, we compared models trained on naturally textured stimuli, versus unnaturally textured “stylized” stimuli [39]. Specifically, we compared performance between ResNet-50 trained on ImageNet, and ResNet-50 trained on stylized images. In order to assess the extent to which the results are driven by the intrinsic architecture of the networks, versus being a consequence of training them for object recognition, we also performed this same analysis on 100 initializations of AlexNet and ResNet-50 with random weights and no training of any kind. The same analysis pipeline was applied to each random initialization independently, and the final results (mean color space correlation across objects within a layer) were averaged to obtain the final result (Fig 2E). Targeted matched-pairs t-tests were used to compare the slopes of these differently-trained networks with the corresponding networks trained on object recognition.

### Quantifying the strength of color representation within each layer

While the previous analyses examined the extent to which the relative similarity structure among different colors varied across objects, this analysis examined a complementary property of the color geometry: the strength of color representation within an object in each CNN layer (Fig 3). To measure this, we computed the average distance among the different colors within each object, using a distant metric that enabled valid comparisons across layers despite varying overall levels of activation and numbers of units in a given CNN layer. Specifically, within an object and layer, we z-normalized the unit responses to each of the 12 colors (equating their mean and variance), computed the pairwise Euclidean distance between each pair of colors, and divided this Euclidean distance by two times the square room of the number of units in order to allow results from layers with varying numbers of units to be compared on the same scale (this was necessary as the distance between two opposite patterns would increase with increasing number of units/dimensions). These pairwise normalized color distances were then averaged across all pairs of colors within each object, and the mean within-object color distances were in turn averaged within each layer to derive an overall measure of how distinctly colors are represented within an object in a given layer. For the random networks, analyses were conducted across 100 random initializations of the networks, with the within-object color distances being averaged at the final step to aggregate results across these initializations.

Several analyses were then performed on these mean within-object color distances. First, for each condition, the mean distance was compared against zero (one-sample t-test) to test for the existence of color information. Second, for each model and layer, the mean distance was compared between the textured and silhouette objects (matched-pairs t-tests) to examine how color information varies based on the presence of texture information. Third, regression analyses were performed to test whether there exists an overall increase or decrease in color processing over the course of each network. This was performed separately for the Textured and Silhouette Objects, and the final (category output) layer was excluded from this analysis. A separate slope was extracted for each object, and a one-sample t-test was used to test whether these slopes significantly vary from zero. Finally, the amount of color information was compared between each layer of the random versions of AlexNet and ResNet-50 (average across 100 random initializations) and the trained versions of these networks, to test whether training the networks for object recognition leads to increased retention of color information.

#### Transformation of color space representations across CNN layers and architectures

To understand how the color space of an object may evolve over the course of processing and whether colors are coded similarly for an object across different layers of a network, for the main original set of 50 objects and 12 colors calibrated in CIELUV color space, we correlated the color space vector for each object in either the first or penultimate layer of each network with its color space vector from each other layer of the network (Fig 4A). These correlation values were then averaged across all objects and plotted in Fig 4B. To test for statistical significance, we performed a regression analysis to examine whether correlations with the first and penultimate layers of the network decrease in layers that are further apart. To do this, for each object, we applied Fisher’s Z transformation to the correlation values, and regressed them onto the positions of the CNN layers (using layer_fraction) of all layers up to, but not including, the comparison layer (first or penultimate layer). A one-sample t-test was then used to test the slopes from all the objects against zero to assess whether the average slope was significantly different from zero. This was done for the networks trained on ImageNet, on 100 initializations of AlexNet with random weights, and 100 initializations of ResNet-50 with random weights.

To examine whether color coding within an object transforms in a similar manner across different networks, for each object we correlated its color space vector from each sampled layer of each network with every other layer (Fig 4C, left column). The resulting similarity space was averaged across objects and visualized using MDS (after subtracting each correlation from 1 to convert to dissimilarity), and the mean pairwise similarities in the first and penultimate layers of each network were reported in S3 Fig.

To examine whether the color space of an object evolves in a similar way in trained networks and in randomly initialized networks, we performed the same analysis as described above comparing the version of AlexNet trained on object recognition with 10 random initializations of AlexNet (Fig 4C, middle column). We also performed the same analysis for ResNet-50, including the ImageNet-trained version, the version trained on stylized images, and ten random initializations (Fig 4C, right column). The mean pairwise similarities in the first and penultimate layers for these comparisons were reported in S4 and S5 Figs.

#### Transformation of color space similarity across CNN layers

To understand how patterns of color space similarities among objects change over the course of processing (i.e., whether the similarity profile among the color spaces of different objects remains consistent over the course of processing), for the main original set of 50 objects and 12 colors calibrated in CIELUV color space, we correlated the color space vector of all objects within each layer with one another to construct a color space similarity RSM for that layer. From the similarity matrix formed, we used the off-diagonal values to define a *color space similarity vector*. The resulting color space similarity vector from the first and penultimate layers were then correlated with those from each of the other layers (Fig 5B).

#### The effect of object form similarity on color space similarity

To understand how color space similarity of two objects is determined by the form similarity of these two objects (Fig 5C) and if two objects with similar forms would also have similar color spaces, for the main set of 50 objects and 12 colors calibrated in CIELUV color space, we first measured the overall object form similarity in each CNN layer for the original set of 50 objects. This was done by calculating all the pairwise Pearson correlations of the CNN layer output to grayscale versions of all the objects. From the similarity matrix formed, we used the off-diagonal values to define an *object form similarity vector*. We then correlated the object form similarity vector with the corresponding color space similarity vector for that CNN layer. The resulting correlation value from each CNN layer was plotted together in a line graph (Fig 5D).

## Supporting information

S1 Fig Color space trajectories of three example objects across every layer of AlexNet. To test whether the layers sampled for the various analyses fully capture the trajectory of color space representation over the course of processing, for three sample objects we computed their color space similarity matrix for *every* layer of processing in AlexNet (trained on ImageNet), computed the second-order dissimilarity matrix among these color space matrices, and visualized the results using MDS. Each trajectory is for a different object, and each ring indicates a different layer (ring colors denote layer type), with the object’s icon being adjacent to the final layer. The layers sampled for the analyses are denoted by solid black dots. The trajectory does not vary based on the type of layer being sampled, as long as they are adjacent to each other in the processing pipeline (e.g., the color space representation of a conv layer is either identical with or adjacent to its representation in any subsequent ReLU or pooling layers), and the sampled layers fully interpolate the overall color space trajectories, with no deviations evident in the intervening unsampled layers.
(TIFF)

S2 Fig Mean between-object color space correlations for each layer and network, using two additional measures. **a.** Same as Fig 2C, but with color space structure of each object measured with Euclidean distance instead of Pearson correlation (similarity *between* color spaces was still calculated with Pearson correlation). Fully-connected layers are marked by hollow circles and other types of layers sampled are marked by solid circles. Results remain qualitatively similar as those in Fig 2C. **b.** Same as Fig 2C, but using colors calibrated in an artificial HSV color space that is not based on human psychophysical judgments. Results again remain qualitatively similar as those in Fig 2C. *** *p* < .001.
(TIFF)

S3 Fig Color space comparisons for the five trained networks. **a.** MDS plots depicting color space correlation across different CNN layers and architectures, plotted separately for the two versions of the objects (copied from Fig 3C for convenience). This was done by constructing a color space correlation matrix for each object including its color space correlation across all sampled layers of all CNNs. The resulting correlation matrix was then averaged across objects and visualized using MDS. Each trajectory is a different network, each dot is a different layer, and the hollow dots denote the first layer of each network. **b.** Exact correlation values for the between network correlations in the first layer of each network (top) and the penultimate layer of each network (bottom) for both versions of the objects (textured objects left, silhouette objects right). Correlations are very similar across networks in the first layer, but diverge by the end of processing.
(TIFF)

S4 Fig Color space comparisons for AlexNet trained with ImageNet images and with 10 different random-weight initializations. **a.** MDS plots depicting color space correlation across different layers for ImageNet trained AlexNet and 10 instances of AlexNet with random-weight initializations, plotted separately for the two versions of the objects (copied from Fig 3C for convenience). **b.** Exact correlation values for the between network correlations in the first layer of each network (top) and the penultimate layer of each network (bottom) for both ImageNet image trained AlexNet and the 10 instances of AlexNet with random-weight initializations, done separately for the two versions of the objects. Other details are the same as S2 Fig. Correlations are very similar across the trained and untrained networks in the first layer and remain similar across all the untrained networks, but differ substantially between the trained and untrained networks by the end of processing.
(TIFF)

S5 Fig Color space comparisons for ResNet-50 trained with ImageNet images, trained with stylized ImageNet images, and with 10 random-weight initializations. a. MDS plots depicting color space correlation across different layers for original ImageNet trained ResNet-50, stylized ImageNet trained ResNet-50, and 10 instances of ResNet-50 with random-weight initializations, plotted separately for the two versions of the objects (copied from Fig 3C for convenience). b. Exact correlation values for the between network correlations in the first layer of each network (top) and the penultimate layer of each network (bottom) for original ImageNet image trained ResNet-50, stylized ImageNet image trained ResNet-50 and the 10 instances of ResNet-50 with random-weight initializations, done separately for the two versions of the objects. Other details are the same as S2 Fig. Correlations are relatively similar across the trained and untrained networks in the first layer, but diverge substantially between the trained and untrained networks, and between the 10 different instances of the untrained networks by the end of processing.
(TIFF)

S1 Table Mean pairwise color space correlations for the 50 textured objects in Alexnet and GoogLeNet trained with ImageNet images and in randomly weighted Alexnet. The mean correlation values are shown for each sampled layer in each network for both the original and the shuffled correlations. For the shuffled correlations, the color labels were randomly shuffled in the color space before a correlation was performed. This was done 100 times for each pair of correlation and the results were averaged.
(DOCX)
