# Supplementary figures and images for: Joint representation of color and form in convolutional neural networks: A stimulus-rich network perspective
Source: PLoS One. 2021 Jun 30;16(6):e0253442. doi: 10.1371/journal.pone.0253442 (PMC8244861; doi:10.1371/journal.pone.0253442)

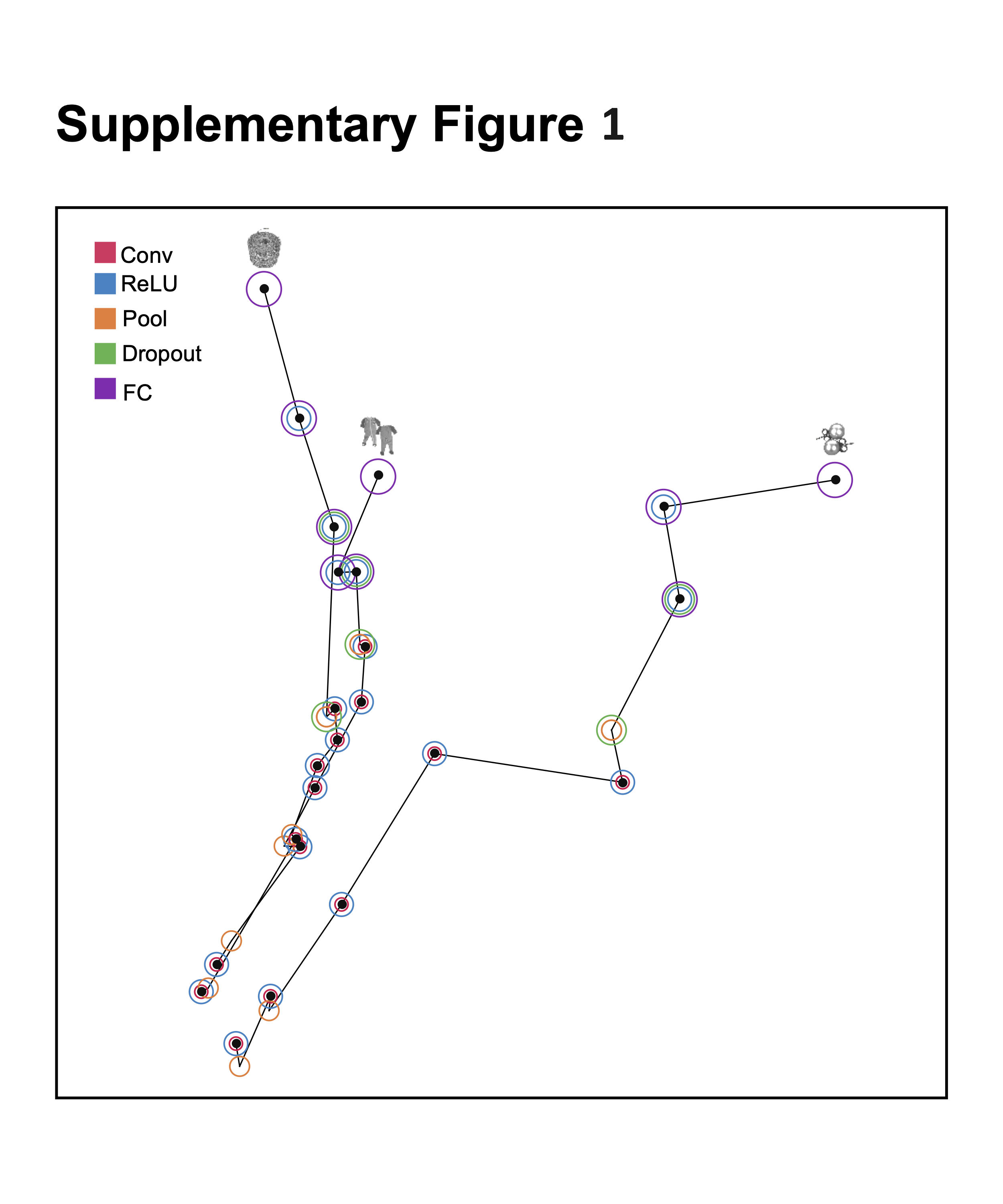

Supplement: S1 Fig — To test whether the layers sampled for the various analyses fully capture the trajectory of color space representation over the course of processing, for three sample objects we computed their color space similarity matrix for every layer of processing in AlexNet (trained on ImageNet), computed the second-order dissimilarity matrix among these color space matrices, and visualized the results using MDS. Each trajectory is for a different object, and each ring indicates a different layer (ring colors denote layer type), with the object’s icon being adjacent to the final layer. The layers sampled for the analyses are denoted by solid black dots. The trajectory does not vary based on the type of layer being sampled, as long as they are adjacent to each other in the processing pipeline (e.g., the color space representation of a conv layer is either identical with or adjacent to its representation in any subsequent ReLU or pooling layers), and the sampled layers fully interpolate the overall color space trajectories, with no deviations evident in the intervening unsampled layers. (TIFF) [file pone.0253442.s001.tiff]

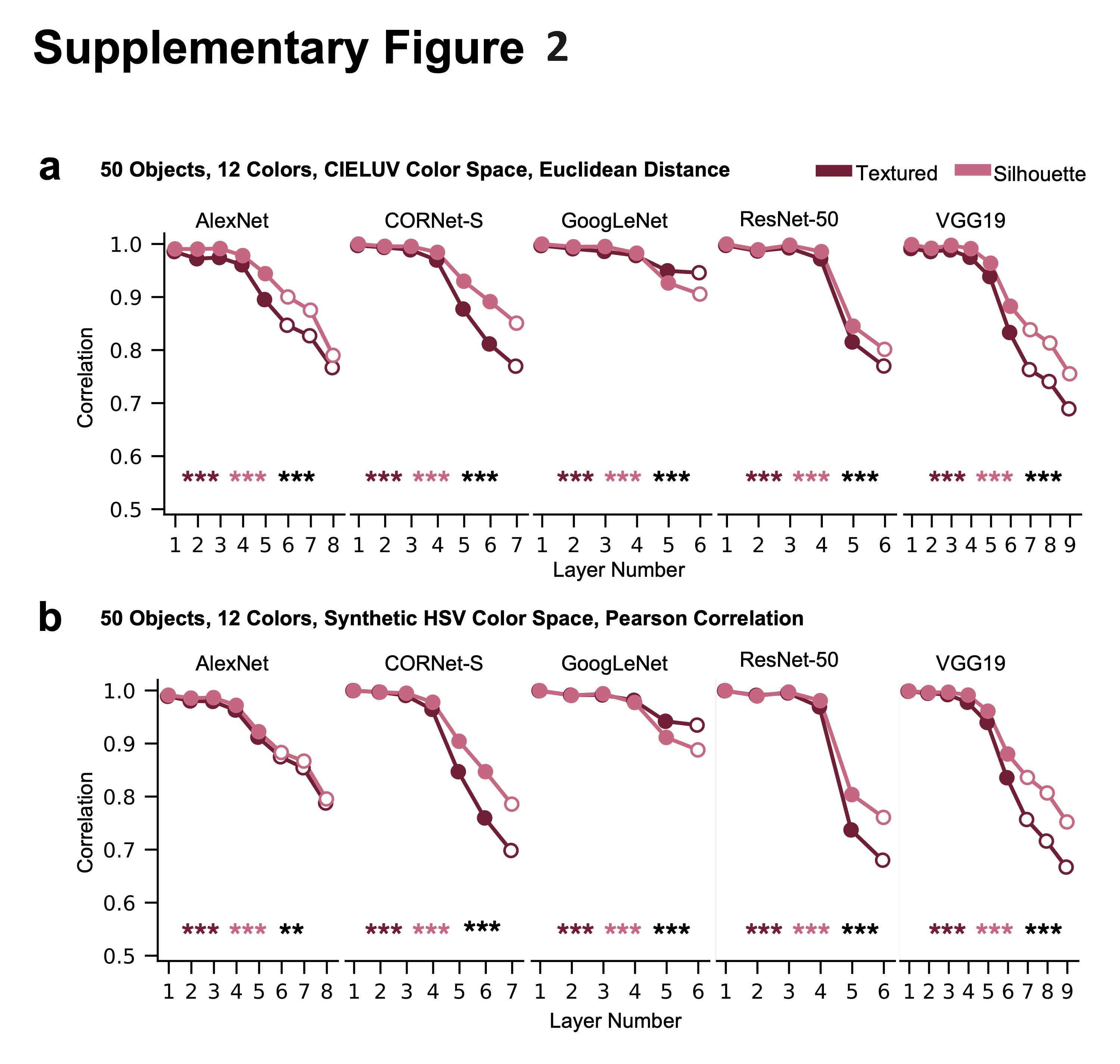

Supplement: S2 Fig — a. Same as Fig 2C, but with color space structure of each object measured with Euclidean distance instead of Pearson correlation (similarity between color spaces was still calculated with Pearson correlation). Fully-connected layers are marked by hollow circles and other types of layers sampled are marked by solid circles. Results remain qualitatively similar as those in Fig 2C. b. Same as Fig 2C, but using colors calibrated in an artificial HSV color space that is not based on human psychophysical judgments. Results again remain qualitatively similar as those in Fig 2C. *** p < .001. (TIFF) [file pone.0253442.s002.tiff]
