# Supplementary material for: Joint representation of color and form in convolutional neural networks: A stimulus-rich network perspective
Source: PLoS One. 2021 Jun 30;16(6):e0253442. doi: 10.1371/journal.pone.0253442 (PMC8244861; doi:10.1371/journal.pone.0253442)
Supplement: S1 Table — The mean correlation values are shown for each sampled layer in each network for both the original and the shuffled correlations. For the shuffled correlations, the color labels were randomly shuffled in the color space before a correlation was performed. This was done 100 times for each pair of correlation and the results were averaged. (DOCX) [file pone.0253442.s006.docx]

| **Model** | **Layer** | **Original Mean Color Space Correlation** | **Resampled Mean Color Space Correlation** |
| --- | --- | --- | --- |
| **AlexNet (ImageNet)** | Conv1 | 0.98 | -0.0003 |
|  | Conv2 | 0.93 | 0.00009 |
|  | Conv3 | 0.94 | 0.00004 |
|  | Conv4 | 0.91 | -0.00002 |
|  | Conv5 | 0.80 | 0.0005 |
|  | FC1 | 0.82 | -0.0003 |
|  | FC2 | 0.79 | 0.0001 |
|  | FC3 | 0.71 | -0.0002 |
| **AlexNet (Random)** | Conv1 | 0.99 | -0.0002 |
|  | Conv2 | 0.99 | -0.0002 |
|  | Conv3 | 0.99 | 0.0002 |
|  | Conv4 | 0.98 | 0.00005 |
|  | Conv5 | 0.98 | -0.0001 |
|  | FC1 | 0.98 | -0.00004 |
|  | FC2 | 0.98 | 0.0001 |
|  | FC3 | 0.98 | 0.0004 |
| **GoogLeNet (ImageNet)** | Conv1 | 0.997 | -0.0001 |
|  | MaxPool2 | 0.99 | -0.0001 |
|  | MaxPool5 | 0.98 | -0.00005 |
|  | MaxPool11 | 0.97 | -0.00004 |
|  | AvgPool1 | 0.94 | -0.0003 |
|  | FC1 | 0.94 | 0.0005 |
